# Oscillatory dynamics of motor learning across adulthood life span: a systematic review

**DOI:** 10.3389/fnagi.2025.1646172

**Published:** 2025-10-20

**Authors:** Amir Jahanian-Najafabadi, Elaheh Davoodi

**Affiliations:** ^1^Department of Cognitive Neuroscience, Bielefeld University, Bielefeld, Germany; ^2^Independent Researcher, Tehran, Iran

**Keywords:** motor learning, EEG oscillations, aging, neuroplasticity, brain connectivity

## Abstract

Motor learning refers to a set of processes associated with practice and experience that are essential for acquiring new skills and adapting behavior throughout the lifespan. Mastery of motor skills plays a crucial role in maintaining autonomy and quality of life, particularly in aging populations. This learning process relies on internal neural mechanisms that lead to enduring changes in movement capability, yet the underlying functional and anatomical adaptations in sensorimotor circuits remain incompletely understood. These adaptations are often influenced by both task characteristics and age, highlighting the need for a deeper understanding of brain activity related to motor learning. In this pre-registered systematic review, we synthesized evidence from experimental studies and randomized controlled trials (RCTs) examining the relationship between motor learning and brain activities, specifically as measured by resting-state and task-related electroencephalography (EEG). We conducted a comprehensive literature search, identifying studies published in English between 2008 and May 2025 from PubMed, Scopus, and Web of Science databases and identified from web pages. After initial screening of 1,910 articles by title and abstract, a total of 80 studies met the eligibility criteria and were included in the final review. Studies were assessed for methodological quality in accordance with PRISMA guidelines. Our review focuses on EEG oscillatory activity across young, middle-aged, and older adults during motor skill acquisition, motor learning, adaptation and motor inhibitory control. We examined whether specific EEG features are linked to predicting motor learning performance, and explored how oscillatory patterns vary by task type, complexity, and age. By integrating findings across diverse studies, this review aims to advance our understanding of the neural mechanisms that support motor learning and its dimensions and inform the development of targeted, age-appropriate empirical research in healthy populations.

**Systematic review registration:** CRD42024569699.

## 1 Introduction

Motor learning involves enduring adjustments to bodily movements in a lifelong process of skill acquisition. This process includes factors such as practice and behavioral modification to overcome challenges encountered while executing actions in response to novel stimuli (Bracha and Bloedel, 2008). These challenges arise from interactions between e.g., the individual, the task, and the environment during motor activities, such as riding a bike or playing an instrument leading to permanent changes in skilled motor behavior (Krakauer et al., 2019; Lee and Schmidt, 2008; Leech et al., 2021; Masaki and Sommer, 2012; Magill and Anderson, 2017; Schmidt, 1988; Schmidt et al., 2018).

One interconnected concept related to motor learning is motor performance. Unlike motor learning, which refers to the long-lasting acquisition, refinement, retention, and improvement of motor skill behavior over time involving cognitive and neural processes, motor performance is defined as the ability to execute a motor task. It requires the integration of muscular and nervous system functions, and reflects the observable outcomes of movement. Motor performance is also a multifaceted state, dependent on distinct performance conditions such as force production, precision control, movement speed, resistance to fatigue, motor adaptation, and, finally, motor learning (Behrens et al., 2022; Forman et al., 2021), which serves as our inclusive term in this systematic review.

Another related concept is motor sequence learning, which refers to the process of acquiring and improving the execution of ordered motor actions through practice and training (Doyon, 2008; Gonzalez and Burke, 2018; Tzvi-Minker, 2015). It enhances both the speed and accuracy of performing learned movements. This type of learning involves predictive processing, allowing individuals to anticipate subsequent movements in a sequence. A key task used to study this phenomenon is the Serial Reaction Time Task (SRTT), which measures response times as participants learn sequences of actions. Brain regions such as the cerebellum and striatum are crucial for automating these tasks, while sequences of varying complexity engage distinct neural circuits. The short-term, immediate enhancements that result from repeated practice highlight practice effects, whereas skill acquisition refers to the comprehensive process of learning a new skill, from initial attempts to achieving proficiency.

Mastering skills through motor learning is underpinned by neurocognitive contributions and neurological processes involving brain activity and synaptic organization and working memory (Constantinidis et al., 2023; Dayan and Cohen, 2011; Mottaz et al., 2024; Rostami et al., 2009; Seidler et al., 2012). Learning-induced functional and anatomical changes within sensorimotor circuits are well-documented. For instance, studies on the explicit learning of sensorimotor tasks, as well as learning within sensory-motor circuits, establish a connection between action and the anticipated result. This suggests that brain states prior to movement can offer insights into the expected success of motor learning (Meyer et al., 2014; Zhou and Schneider, 2024).

Both cortical and subcortical regions contribute significantly to motor sequence learning, with cerebro-cortical and striatal-cortical networks playing a key role (Hikosaka et al., 2002; Doyon et al., 2002; Tzvi et al., 2014; Penhune and Doyon, 2002). Understanding these connections is essential for comprehending the mechanisms underlying motor learning, which is crucial for advancing motor learning enhancement and neurorehabilitation practices. However, due to genetic factors and individual differences in brain structure and function, the neurophysiology of motor learning remains not fully understood, whereas gaining knowledge in this area even can help predicting motor performance from resting neural markers (Tomassini et al., 2010; Herszage et al., 2020; Williams and Gross, 1980).

Several theoretical frameworks shaped our perception of motor skill acquisition and learning, such as Fitts and Posner Three-Stage Model, Bernstein's Degrees of Freedom Model, Gentile's Two-Stage Model, and Schmidt's Schema Theory. Contemporary frameworks and Modern Computational Models describe different motor learning mechanisms mapped onto specific neural regions, which are key for motor skill acquisition (Bernstein, 1966; Cano-de-la-Cuerda et al., 2015; Fitts, 1967; Gentile, 1972; Leech et al., 2022). What is less known and partially understood is the neural circuits engaged during skill acquisition that are modulated specifically by practice-based performance improvement, and those that predict recall performance. Moreover, the growing evidence suggests that brain activity during practice in the primary motor cortex and basal ganglia is associated with trial-by-trial practice performance which is predictive of immediate recall performance. These frameworks offer distinct perspectives on how new motor skills are acquired and refined, highlighting the need for further research to unravel how these neural activities translate into long-term skill retention, and expertise (Beroukhim-Kay et al., 2022; Cano-de-la-Cuerda et al., 2015).

Previous studies have highlighted the considerable roles of various brain structures in motor learning and skill acquisition. The prefrontal cortex (PFC) is involved in the cognitive processes required for mastering new motor skills (Grafton and Volz, 2019; Tian and Chen, 2021; Friedman and Robbins, 2021). The cingulate cortex facilitates motor control, error detection, performance evaluation, and the refinement of motor skills (Asemi et al., 2015; Paus, 2001). The primary motor cortex (M1) contains a somatotopic motor map corresponding to specific body part movements, which undergoes neuroplastic changes to accommodate new skills and enhance existing ones (Kandel et al., 2013; Papale and Hooks, 2017; Seidler, 2009; Tian and Chen, 2021). The supplementary motor area (SMA) is more involved in internally-generated movements is interconnected with M1 (Hardwick et al., 2012; Welniarz et al., 2019), the dorsal premotor cortex (PMd), and the ventral premotor cortex (PMC), facilitating the guidance of motor actions through sensory input especially externally-guided movements (Hoshi and Tanji, 2007; Kantak et al., 2011).

The basal ganglia and cerebellum support motor learning through complementary mechanisms that enable smooth, coordinated initiation and maintenance of movement (Baladron et al., 2023; Doyon et al., 2009; Torbati et al., 2024). The hippocampus also interacts with the striatum during motor sequence learning (Albouy et al., 2008, 2014). The coordinated activity of the primary somatosensory cortex (S1) and the posterior parietal cortex (PPC) supports motor movements through both somatosensory and visual feedback (Mirdamadi et al., 2025; Wang et al., 2024). Furthermore, major functional and anatomical networks including the basal ganglia, cerebellum, M1, SMA, premotor cortex, sensorimotor cortex, parietal cortex, right thalamus, cingulate gyrus, and putamen have been associated with motor deficits, spatial and sensorimotor learning, and motor sequence learning (Lefebvre et al., 2012; Penhune and Steele, 2012).

### 1.1 Age-related motor learning

Aging is associated with reductions in gray matter volume in key brain areas, including the primary motor cortex, somatosensory cortex, and cerebellum. These structural and functional brain changes can impact movement speed, coordination, and precision (Good et al., 2001; Salat, 2004; Seidler, 2009; Ward, 2003). Even in the absence of neurodegenerative disease, aging is characterized by alterations in sensorimotor activity, resource allocation, and cognitive-motor interactions, all of which affect perception, movement, and cognition (Seidler, 2009). While sensorimotor function declines with age, training and brain stimulation techniques have the potential to modulate these effects. Several studies have reported age-related declines in motor performance and motor learning (Brown et al., 2009; Durkina et al., 1995; Janacsek and Nemeth, 2012; Shimoyama, 1990). For example, simple repetitive tasks such as finger tapping show a reduction in frequency with age (Shimoyama, 1990). Likewise, older adults exhibit reduced learning rates in tasks like the pursuit of motor learning across multiple days (Durkina et al., 1995). Although immediate learning gains are often observed, the consolidation of motor memory is particularly affected by age (Janacsek and Nemeth, 2012).

Prior research has highlighted the importance of age-related differences in motor learning capacity and performance, often estimated through specific neural oscillatory bands such as mu and beta (Deiber et al., 2014; Liu et al., 2017; Rueda-Delgado et al., 2019). Few studies have directly investigated the impact of age on brain oscillations as predictors of motor learning improvements. However, existing evidence suggests that age-related changes in brain function, cognition, and motor abilities likely interact to influence learning outcomes (Espenhahn et al., 2019; Wang et al., 2019). Understanding these effects is critical for supporting functional independence in aging populations and for developing personalized sensorimotor training and rehabilitation protocols. This highlights the need to incorporate age, neural oscillatory patterns, and cognitive measures into motor learning research to capture a more comprehensive picture of individual differences across the lifespan.

Aging studies have consistently shown reduced processing efficiency, accompanied by declines in working memory and slower response times (Berchicci et al., 2012; Hedden and Gabrieli, 2004). Older adults generally require more cognitive resources for planning and executing motor tasks compared to younger individuals. Additionally, slower information processing and reduced attentional capacity can further hinder (Seidler, 2009). Given the centrality of aging in this context, this systematic review aims to examine how motor learning is shaped by age-related neural changes, specifically through EEG-measured oscillatory brain activity. We focus on motor skill acquisition and adaptation across the adult lifespan, highlighting neural patterns that may predict learning outcomes and inform individualized interventions for age-related motor deficits.

Examining both resting-state and task-related brain activities in the context of motor learning offers insight into how aging affects the ability to acquire and retain new motor skills. However, the precise effects of aging on motor skill acquisition, retention, and neural plasticity following practice remain inconclusive and merit further investigation. In this line, studies have shown that age affects both motor learning and associated alpha activity. It has been reported that the neural circuits involved in motor skill acquisition in older adults are like those in younger individuals, but older adults tend to exhibit more widespread activation patterns. This suggests that while the same networks are engaged, the efficiency of their use may differ due to age-related changes (Bootsma et al., 2021; Berghuis et al., 2019). There is also another study that investigated the effects of alpha-wave binaural acoustic beats on motor learning across different age groups. Their findings suggested that this type of stimulation could improve motor performance in older adults by enhancing alpha activity, thereby influencing their learning processes differently than in younger individuals (Herozi et al., 2024; Durand-Ruel et al., 2023). According to Park et al. (2025), resting-state oscillations are associated with age. The findings indicated that decreased alpha and altered beta activity with age provide foundational insights that relate to age-related changes in neural oscillations vary as a function of brain region and frequency band. Since these oscillations are known to influence neuroplasticity and motor performance, that is interesting to see how such brain oscillations change with age, which could influence motor learning processes (Park et al., 2025).

In this review, we synthesized current EEG research to better understand how age shapes motor skill acquisition, with a focus on oscillatory dynamics and neural plasticity. Specifically, we examined findings from studies using EEG to assess both resting-state and task-related activity, focusing on how age-related changes in e.g., alpha, beta, and mu rhythms relate to learning processes. By integrating results across studies, we aim to clarify how neural oscillations and functional connectivity evolve with age and how these changes shape the capacity to acquire and retain new motor skills. This approach allows for a more comprehensive understanding of the neural mechanisms underlying motor learning across the lifespan. Furthermore, it reveals important gaps in the literature, particularly in relation to age-specific variability in training outcomes. Additionally, these efforts would underscore the need for personalized approaches to motor rehabilitation and cognitive-motor interventions in older adults. In this context, the present review not only highlights patterns of compensatory brain activity associated with aging but also sets the stage for future research aimed at optimizing motor learning strategies through targeted neurophysiological markers.

### 1.2 Brain oscillations as a signature of motor learning

In recent decades, electroencephalography (EEG) has emerged as a non-invasive technique to measure neurological activity associated with motor tasks, offering insights into the brain mechanisms involved in the learning and adaptation of motor skills (Ahmadian et al., 2013; Hamada et al., 2023; Haar and Faisal, 2020a,b; Jee, 2021). Brain waves are classified into delta (0.5–4 Hz), theta (4–8 Hz), alpha (8–12 Hz), beta (13–30 Hz), and gamma (>30 Hz) frequency bands using the Fast Fourier Transform (FFT) technique. Each EEG frequency band is associated with distinct psychophysiological states. Studies suggest a correlation between EEG frequency band activity and the neural mechanisms underlying motor learning and task success (Teplan, 2002; Nayak and Anilkumar, 2025).

However, the results of individual studies remain inconclusive when considered alone. Across the literature, six EEG frequency bands have been associated with motor learning. Among these, beta (13–30 Hz) (Espenhahn et al., 2019), alpha (8–12 Hz) (Ghasemian et al., 2016), and theta (5–8 Hz) (Van Der Cruijsen et al., 2021) have been consistently identified as the most relevant for predicting and understanding motor actions. In addition, gamma (30–100 Hz) (Amo et al., 2015; Amo et al., 2017; Usanos et al., 2020), delta (2–4 Hz) (Hamel-Thibault et al., 2016; Wong et al., 2013), and mu bands (8–13 Hz) (Nakayashiki et al., 2014; Deiber et al., 2014; Zhang and Fong, 2019) are believed to offer valuable insights into predicting motor learning outcomes. These insights come from analyses of both task-based activity and resting-state functional connectivity (rsFC) (Sugata et al., 2020; see Table 1 for details).

**Table 1:** Age-related findings across studies.

**No**.	**References**	**Age group**	**Frequency band**	**Task type**	**Learning phase**	**Key findings**
1	Babaeeghazvini et al. (2018)	Young & old adults	• Beta band	• A bimanual visuomotor task	• Focused on motor performance at baseline/rest and its association with age differences in connectivity rather than specific motor learning phases.	• Older adults showed increased beta-band functional connectivity (wPLI values) particularly in the left intra-hemispheric pathway between the dorsal premotor area (PMd L) and primary motor cortex (M1 L). • In younger adults, different relationships were observed linking structural and functional connectivity in the beta band with motor performance, but the key beta-band difference with aging was this increased functional connectivity at rest in left intra-hemispheric and inter-hemispheric motor areas, associated with motor decline.
2	Bayram et al. (2015)	Young & old adults	• Beta band (15–35 Hz)	• Maximal Voluntary Contraction (MVC) • Submaximal Elbow Flexion (EF) at 20%, 50%, and 80% MVC	• Focused on motor	• Older adults showed weakened corticomuscular coherence (CMC) compared to young adults across all force levels. • The decline in CMC may contribute to motor impairment and muscle weakness in aging.
3	Bayram et al. (2023)	Young & old adults	• Beta band	• Voluntary movement task • Voluntary elbow flexion (EF)	• Focused on the execution phase of the motor task and the graded force levels	• The elderly showed significantly lower total EEG spectral power (ESP) at high force (80% MVC) compared to young. • The failure of beta-band relative ESP to decrease with increasing force in the elderly is suggested as a potential biomarker for age-related motor control decline.
4	Bönstrup et al. (2015a)	Young & old adults	• Upper Alpha band (12–14 Hz)	• A complex finger-tapping sequence	• Assessed cortical activity related to execution and suppression (inhibition) of a motor skill in the post-learning phase, emphasizing motor memory retrieval and context-sensitive inhibition rather than initial acquisition or early learning	• Aging is associated with reduced alpha-band activity during motor inhibition of a well-learned motor skill after consolidation, indicating deficits in inhibitory cortical mechanisms with age and altered plasticity following motor learning.
5	Bootsma et al. (2021)	Young & old adults	. • Alpha & beta bands	• Mirror star tracing task at one of three difficulty levels.	• Motor skill acquisition and retention phases.	• Both age and task difficulty influence motor learning and its neural correlates in alpha and beta EEG bands, with older adults exhibiting more bilateral cortical activity and differing retention depending on difficulty.
6	Chettouf et al. (2022)	Young & old adults	• Beta band	• Unimanual motor task	• Acquisition and within-session practice.	• Aging reduces the magnitude and beta-band motor oscillations during unimanual motor learning, despite largely preserved task-related hemodynamic responses
7	Christov and Dushanova (2016)	Young & old adults	• Beta & Gamma bands (β1: 12.5–20; 12.5–20; β2: 20.5–30 Hz) (γ1: 30.5–49; γ2: 52–69 Hz)	• Binary choice-reaction sensorimotor task	• Analyzed during sensory (early) and cognitive (late) processing phases after stimulus onset	• Age-related alterations in beta and gamma oscillatory dynamics predominantly during cognitive processing after auditory stimulation, with task-related motor responses assessed during sensory and early cognitive response phases
8	Deiber et al. (2014)	Young & old adults	• Mu (9–12 Hz) & Low Beta (15–20 Hz)	• Three delayed motor tasks	• Motor preparation phase	• Age-related reductions in the lateralization of mu and beta oscillations during internal motor preparation, highlighting deficits in free movement selection mechanisms in older adults
9	Espenhahn et al. (2019)	Young & old adults	• Alpha & Beta (15–30 Hz)	• Mirror drawing a novel wrist flexion/extension tracking task & subsequently retested at two different time points (45–60 min and 24 h after initial training).	• Motor task was assessed during the acquisition and early retention phases	• Movement-related beta desynchronization (MRBD) in the ipsilateral sensorimotor cortex before training predicted individual performance 45–60 min after training. • Cortical beta-band oscillations, especially movement-related desynchronization, are important neural markers that explain variability in short-term motor learning performance in both young and elderly
10	Frolov et al. (2020)	Young & old adults	• Mu, beta &theta bands	• A fine motor task involves squeezing one of their hands into a fist after an audio signal and holding it until a second signal. They performed this task 30 times with each hand, for a total of 60 repetitions	• Motor initiation (preparation) phase	• Elderly adults display slowed motor initiation linked to increased theta and altered mu/beta band dynamics during the motor preparation phase
11	Herozi et al. (2024)	Young & old adults	• Alpha band	• Digital mirror-tracing task	• The motor task was assessed in the acquisition and practice phase of motor learning.	• Alpha band stimulation for 30 min led to a significant decrease in errors in older adults but not in young adults. • In younger adults, alpha band significantly decreased reaction time but did not reduce errors.
12	Jahanian et al. (2023c)	Young & old adults	• Beta band,	• Dart skill learning • Dart throwing using both dominant and non-dominant hands.	• Assessed spans acquisition through practice-induced changes	• Greater beta relative power predicts stronger Practice effect in younger adults • Lower Beta relative power predicts stronger practice effect in older adult
13	Liu et al. (2017)	Young & old adults	• Alpha & beta, bands	• Finger task	• Focused on movement execution and preparation phases	• Older adults exhibited a reduction in beta power suppression during movement compared to younger adults.
14	Lum et al. (2023b)	Young & middle aged	• Theta, alpha & beta bands	• Serial Reaction Time task; unknowingly repeat a sequence of finger movements in response to a visual stimulus	• Acquisition (implicit learning) phase	• Lower upper theta resting-state EEG power predicts greater implicit motor sequence learning ability, with the motor task assessed during the acquisition phase
15	Penalver-Andres et al. (2022)	Young to old adults	• Alpha band	• Virtual surfing, requiring participants to steer a virtual boat using a joystick to surf waves as quickly as possible to a finish line following a resting-state EEG recording alternating between EO & EC conditions	• The resting brain state as a trait marker predictive of acquisition and motor performance stage	• Resting-state functional network activity, especially posterior DMN preactivation, predicts motor performance in a complex visuomotor task. • Age & gaming/sailing experience were not significant confounding factors in the main findings
16	Rueda-Delgado et al. (2019)	Young & old adults	• Alpha & beta bands	• A bimanual coordination task	• Placed the assessment primarily in the acquisition and early consolidation phases of learning.	Both groups improved motor performance with practice, but older adults showed less learning. • Beta power (15–30 Hz) decreased more with training in young adults, especially in sensorimotor cortices, indicating more flexible neural reorganization. • Older adults showed a smaller beta power decrease and altered alpha (8–12 Hz) band modulation after training, reflecting less neural plasticity.
17	Sallard et al. (2014)	Young & old adults	• Low Beta (14–20 Hz), & High Beta (20–30 Hz) bands	• Unimanual Tapping (UM) & Bimanual Tapping (BM)	• Evaluated brain activity during motor execution rather than motor learning stages.	• Altered low beta-band oscillatory patterns and behavioral deficits in bimanual coordination in elderly compared to young adults, linked to reduced sensory reafference processing, assessed during self-paced motor execution rather than a specific motor learning stage
18	Veldman et al. (2021)	Young & old adults	• Alpha & beta band	• Visuomotor task consisted of following a template using right- & left wrist flexion & extension movements	• Evaluated motor skill acquisition (immediately after practice), consolidation (retention 24 h later), and interlimb transfer (performance in the untrained left hand).	• Beta-band motor network connectivity modulation is critical for these motor learning mechanisms. • Older adults may use distinct neural connectivity patterns during learning and transfer.
19	Vieluf et al. (2018)	Young & old adults	• Alpha band (8–13 Hz) and also low beta beta (13–20 Hz), high beta (20–30 Hz), and theta (4–8 Hz) bands	• Force maintenance task:Precision grip task	• Assessed expertise differences during a force control motor task	• Young novices had decreased alpha magnitude compared to old groups. • High beta magnitude was lower in old novices compared to young novices and older experts. • Young novices showed higher low beta magnitude for their left hand, whereas old experts showed higher beta magnitude for the right hand. • Attentional network activation was lower in old experts compared to novices, suggesting different control strategies.
20	Yordanova et al. (2020)	Young & old adults	• Theta band (3.5–7 Hz)	• Four-choice reaction task (CRT)	• The motor task assessed response execution	• Older adults displayed a strong theta power at the medial fronto-central region, which correlated with response speed only in young adults, indicating an aging-related dysfunction in medial frontal theta mechanisms.
21	Yordanova et al. (2024)	Young & old adults	• Theta band	• Simple Reaction Task (SRT) • Go-NoGo Task • Four-Choice Reaction Task (CRT) in two modalities, auditory & visual	• Focused on sensorimotor reaction execution	• Older adults showed altered functional connectivity in the theta band during sensorimotor reactions, relying more on sensorimotor feedback during movement execution, compensating for reduced cognitive regulation of motor areas, compared to younger adults.
22	Quandt et al. (2016)	Young & old adults	• Alpha (8–13 Hz) & beta (13–25 Hz) bands	• Finger sequence task (learning and executing a digit sequence). Pinch grip and whole hand grip task (repetitive gripping).	• Participants completed the acquisition phase prior to the EEG recording. • EEG session assessed neural activity during execution of a well-learned (post-acquisition) motor sequence	• Age differences in power decrease were significant mostly in the low beta frequency range (13–19 Hz), where elderly had larger broadband power decreases and a wider recruitment of the motor network. • Elderly showed higher spectral entropy in sensorimotor and frontal regions, indicating a more variable, less sharply tuned motor oscillatory response compared to younger adults.
23	Ricci et al. (2019)	Young & old adults	• Beta band	• 30-min center-out reaching task: move a cursor with right hand to targets appearing randomly on screen using a digitizing tablet. 840 target presentations (15 sets of 56 each)	• Practice/acquisition during movement	• Both groups showed a similar increase in beta modulation depth with practice in both the left and the frontal ROIs.

EEG power spectral density (PSD)—considered an indicator of motor learning which is linked to a range of brain functions. Spontaneous brain activity and PSD contribute to the encoding of information during motor learning, with alpha and beta bands specifically associated with motor performance (Hamada et al., 2023; Livne et al., 2022). In addition, PSD provides a robust framework for examining the neural correlates of motor learning and task success, making it an effective tool for understanding brain activity during motor tasks.

Research has demonstrated that frequency bands such as theta, alpha, beta, and gamma play distinct roles in visual attention and motor memory, indicating that PSD effectively captures these variations (Aliakbaryhosseinabadi et al., 2021; Hamada et al., 2023).

Beta oscillations are particularly sensitive to components of motor tasks involving top-down processing and sensorimotor behavior (Barone and Rossiter, 2021; Engel and Fries, 2010). These oscillations play a central role in motor learning, especially through their engagement with the primary motor cortex (M1) and brain connectivity with other brain regions. Beta-band activity has been shown to correlate closely with motor execution, preparation, and learning (Sugata et al., 2020). Notably, beta-band resting-state functional connectivity (rs-FC) predicts motor learning ability, with stronger beta connectivity between M1 and other areas correlating with better learning outcomes.

Beta activity is sensitive to the motor components of tasks. The so-called beta rebound, which is an increase in beta power exceeding resting levels and is a known marker of movement termination (Studer et al., 2010). During motor learning, average beta activity tends to decrease, while beta modulation related to motor tasks increases. This is accompanied by more pronounced synchronization/desynchronization volleys (Houweling et al., 2009). Baseline beta levels have been identified as predictors of subsequent learning and consolidation processes (Titone et al., 2022). For instance, a single session of practicing a pursuit-tracking motor skill was shown to reduce beta coherence between FC and Cz electrodes in young adults (Ghasemian et al., 2017).

Studies also suggested that cortical electrical activity is involved in movement execution during motor performance (Espenhahn et al., 2019; Jahanian et al., 2023c). Following visuomotor learning tasks, movement-related beta activity has been found to predict individual performance by 1 h, but not 24 h, after training (Espenhahn et al., 2019). Changes in beta-band connectivity during and after motor tasks are associated with motor memory consolidation, indicating that beta oscillations play a vital role in stabilizing newly acquired skills. However, individual variability exists as prior research revealed that higher baseline beta connectivity may correlate with poorer motor learning and weaker consolidation outcomes (Titone et al., 2022).

The stabilization of newly learned motor skills appears to rely on beta-band connectivity changes during and after learning akin to capturing and preserving a photograph of the learned movement for future recall. Yet, as with each musician in a band having unique strengths and weaknesses, individuals with higher baseline beta connectivity may sometimes face challenges in motor learning and adaptation due to the inherent characteristics of their neural networks (Watson, 2006; Aliakbaryhosseinabadi et al., 2021; Özdenizci et al., 2017; Peng et al., 2024). Overall, while beta-band oscillations are essential for motor learning, their specific roles may vary depending on the neural networks involved and individual differences.

Recent studies have also highlighted the importance of alpha activity patterns in motor learning. Alpha oscillations can influence the acquisition, retention, and efficiency of motor preparation (Ghasemian et al., 2016; Deiber et al., 2014).

Within motor sequence learning networks, functional decoupling in the motor-cerebellar loop has been observed by changes in alpha coherence between the premotor cortex and the cerebellum. Moreover, alpha activity has been shown to predict up to 60% of the variability in perceptual learning outcomes (Sigala et al., 2014; Schubert et al., 2020). These findings carry significant implications for rehabilitation strategies aimed at enhancing motor skills in individuals with movement disorders. Interventions such as neurofeedback targeting alpha suppression may be employed to increase cortical excitability and facilitate improved learning outcomes (Wan et al., 2014).

Theta oscillations (4–6 Hz) have been further shown to influence key aspects of motor skill acquisition, retention and are strongly associated with successful motor learning outcomes (Van Der Cruijsen et al., 2021). Additionally, Akkad et al. (2021) reported that enhanced motor skill acquisition was associated with increased theta-gamma phase-amplitude coupling, indicating that this can enhance non-hippocampal motor learning.

Although brain oscillatory activity has given useful understanding in measuring, and predicting motor learning outcomes across various tasks, less is known about the time course of training-related neural changes in alpha, beta, and theta bands, and how these changes interact with specific training parameters and moreover emphasizing brain network dynamics and inter-regional communication provides a clearer and more powerful understanding and prediction of motor learning and motor sequence learning outcomes than considering oscillatory activity alone (Dyck and Klaes, 2024; Mottaz et al., 2024; Takeuchi and Izumi, 2021).

Moreover, resting-state networks offer key insights into aging-related changes in brain dynamics. It is hypothesized that meaningful insights into predicting motor learning outcomes can be gained through the analysis of both task-based and rsFC. Despite numerous studies, questions remain about whether resting-state and task-related brain oscillations are linked to motor learning and can reliably predict short- and long-term effects of motor learning, and how these effects may vary with age. Prior research has shown that resting-state EEG can successfully predict motor learning in both clinical and healthy populations by characterizing baseline brain states and relating them to behavioral variability (Wu et al., 2014; Penalver-Andres et al., 2022). Thus, it is essential to examine whether resting-state EEG power can account for interindividual differences in motor performance and learning (Hübner et al., 2018; Imani and Godde, 2024a,b; Jahanian et al., 2023c; Özdenizci et al., 2016). The relationship between EEG activity at rest and during/after motor task execution can provide valuable insights into motor learning and motor sequence learning (Dyck and Klaes, 2024; Takeuchi and Izumi, 2021). Notably, there have been few studies regarding high alpha amplitude at rest that could predict alpha neuromodulation (e.g., alpha neurofeedback) has been found to predict learning success (Chikhi et al., 2023; Wan et al., 2014).

### 1.3 Current systematic review

Aging is associated with progressive changes in both cognitive and motor functions, which can significantly impact an individual's ability to learn and retain motor skills (Ren et al., 2013). Understanding the neural mechanisms that support motor learning across the adult lifespan is therefore critical, particularly in the context of designing interventions for age-related motor decline. The primary objective of this systematic review is to examine age-related differences in EEG-measured brain activity during motor learning. Specifically, we investigate how patterns of neural oscillations differ among young adults (18–35 years), middle-aged adults (35–55 years), and older adults (55–85 years) during motor skill acquisition, learning, and adaptation. By comparing these age groups, we aim to identify how aging influences the cortical dynamics that underlie motor learning processes.

This review focuses on studies utilizing both resting-state and task-related EEG recordings to capture oscillatory brain activity associated with motor performance. We seek to determine whether specific features of EEG oscillations measured either before or during training can predict individual learning outcomes. A further aim is to explore whether these neural signatures vary with task complexity and learning phase, and whether such changes are age-dependent.

We address several core questions:

- How do brain oscillations during motor skill acquisition correlate with training performance outcomes?
- Are resting-state and task-evoked brain oscillations associated with motor learning ability, and can they serve as predictors of learning outcomes?
- Do changes in cortical activity vary with task difficulty, and are they linked to training effects and the paradigms?
- Finally, are these neural changes and their relationships with motor learning age-dependent?

In addition, we examined whether changes in brain oscillations are task-specific and how neural activity adapts with increasing task complexity across different age cohorts. By synthesizing findings across these dimensions, this review aims to offer a comprehensive account of how motor learning is supported by EEG-measured brain activity throughout adulthood. Ultimately, this work addresses key gaps in the literature by integrating evidence on neural oscillations and motor learning across the adult lifespan. The insights gained are expected to advance our understanding of the neurophysiological basis of age-related motor learning differences and inform the development of tailored rehabilitation and training strategies for older adults experiencing motor impairments.

## 2 Materials and methods

### 2.1 Study selection and data collection

This systematic review has followed the standards of the PRISMA statement (Page et al., 2021a,b). To address the research questions, we considered the PICO format (Urrútia and Bonfill, 2010), and the review was prospectively registered on PROSPERO with the identification number CRD42024569699.

### 2.2 Search strategy

Authors independently searched the databases to find the relevant studies, using the PubMed, Scopus and Web of Science databases, and using the search terms in English (“EEG” OR “Electroencephalography ”) AND (“Brain oscillations” OR “brain networks” OR “rest state” OR “resting-state EEG” OR “task-based EEG”) AND (“Motor learning”) AND (“older adult” OR “young adult” OR “aging”).

### 2.3 Eligibility criteria

Studies were included if they met the following criteria:

(a) published in English, (b) published between 2008 and 2025, (c) the subjects of study were healthy young and older adults within the age range of 18–80, (d) the original RCT or experimental studies with parallel groups or cross over designs; (e) the intervention was single and multisession exercise; motor training, motor learning, or motor activity, motor planning, motor inhibitory control, motor performance improvement; and (f) the outcome was EEG activities in different age groups and based on different motor learning tasks. Studies were excluded if the language was non-English, case report studies, clinical trials, animal studies, or studies that used non-motor training interventions such as drugs or brain stimulation techniques. The search strategy for each used database is presented in Boxes 1–3.

Box 1 **Search strategy for PubMed.**

(“EEG” OR “Electroencephalography “) AND (“alpha” OR “alpha wave “ OR “alpha frequency” OR “alpha band activity” OR “alpha power” OR “alpha coherence” OR “alpha oscillations” OR “beta” OR “beta wave” OR “beta frequency” OR “beta band activity” OR “beta power” OR “beta coherence” OR “beta oscillations” OR “gamma” OR “gamma wave” OR “gamma frequency” OR “gamma band activity” OR “gamma power” OR “gamma coherence” OR “gamma oscillations” OR “delta” OR “delta wave” OR “ delta wave “ OR “delta frequency” OR “delta band activity” OR “delta power” OR “delta coherence” OR “delta oscillations” OR “theta” OR “theta wave” OR “theta wave “ OR “theta frequency” OR “theta band activity” OR “theta power” OR “theta coherence” OR “theta oscillations” OR “mu” OR “mu wave” OR “mu frequency” OR “mu band activity” OR “mu power” OR “mu coherence” OR “mu oscillations” OR “power spectrum” OR “power spectra density” OR “Brain oscillations” OR “brain networks” OR “coherence” OR “rest state” OR “rest-state” OR “resting-state” OR “resting-state EEG” OR “resting-state functional connectivity” OR “resting state power” OR “resting-state power” OR “task-based” OR “task-based EEG” OR “task related” OR “task-related” OR “task related power” OR “task-related power” OR “synchronization”[tw] OR “task related synchronization”[tw] OR “task-related synchronization”[tw] OR “task related desynchronization” OR “task-related desynchronization”) AND (“Motor learning” OR “Motor control” OR “Motor sequence learning” OR “Motor sequential learning” OR “Sensorimotor learning” OR “Motor imagery” OR “ Kinematics” OR “Motor training” OR “Motor practice” OR “Motor exercise” OR “Motor expertise” OR “Motor task” OR “skill acquisition” OR “Motor cortex”) AND (“older adult” OR “young adult” OR “aging”).

Box 2 **Search strategy for SCOPUS.**

TITLE-ABS-KEY ((“EEG” OR “Electroencephalography”)) AND TITLE-ABS-KEY ((“alpha” OR “alpha wave “ OR “alpha frequency” OR “alpha band activity” OR “alpha power” OR “alpha coherence” OR “alpha oscillations” OR “beta” OR “beta wave” OR “beta frequency” OR “beta band activity” OR “beta power” OR “beta coherence” OR “beta oscillations” OR “gamma” OR “gamma wave” OR “gamma frequency” OR “gamma band activity” OR “gamma power” OR “gamma coherence” OR “gamma oscillations” OR “delta” OR “delta wave” OR “ delta wave “ OR “delta frequency” OR “delta band activity” OR “delta power” OR “delta coherence” OR “delta oscillations” OR “theta” OR “theta wave” OR “theta wave “ OR “theta frequency” OR “theta band activity” OR “theta power” OR “theta coherence” OR “theta oscillations” OR “mu” OR “mu wave” OR “mu frequency” OR “mu band activity” OR “mu power” OR “mu coherence” OR “mu oscillations” OR “power spectrum” OR “power spectra density” OR “Brain oscillations” OR “brain networks” OR “coherence” OR “rest state” OR “rest-state” OR “resting-state” OR “resting-state EEG” OR “resting-state functional connectivity” OR “resting state power” OR “resting-state power” OR “task-based” OR “task-based EEG” OR “task related” OR “task-related” OR “task related power” OR “task-related power” OR “synchronization” OR “task related synchronization” OR “task-related synchronization” OR “task related desynchronization” OR “task-related desynchronization”)) AND TITLE-ABS-KEY ((“Motor learning” OR “Motor control” OR “Motor sequence learning” OR “Motor sequential learning” OR “Sensorimotor learning” OR “Motor imagery” OR “ Kinematics” OR “Motor training” OR “Motor practice” OR “Motor exercise” OR “ Motor expertise” OR “Motor task” OR “skill acquisition” OR “Motor cortex”)) AND TITLE-ABS-KEY ((“older adult” OR “young adult” OR “aging”)) AND PUBYEAR > 2007 AND PUBYEAR > 2007 AND PUBYEAR < 2025.

Box 3 **Search strategy for WEB of SCIENCE.**

TS=(“EEG” OR “Electroencephalography”) AND TS=(“alpha” OR “alpha wave “ OR “alpha frequency” OR “alpha band activity” OR “alpha power” OR “alpha coherence” OR “alpha oscillations” OR “beta” OR “beta wave” OR “beta frequency” OR “beta band activity” OR “beta power” OR “beta coherence” OR “beta oscillations” OR “gamma” OR “gamma wave” OR “gamma frequency” OR “gamma band activity” OR “gamma power” OR “gamma coherence” OR “gamma oscillations” OR “delta” OR “delta wave” OR “ delta wave “ OR “delta frequency” OR “delta band activity” OR “delta power” OR “delta coherence” OR “delta oscillations” OR “theta” OR “theta wave” OR “theta wave “ OR “theta frequency” OR “theta band activity” OR “theta power” OR “theta coherence” OR “theta oscillations” OR “mu” OR “mu wave” OR “mu frequency” OR “mu band activity” OR “mu power” OR “mu coherence” OR “mu oscillations” OR “power spectrum” OR “power spectra density” OR “Brain oscillations” OR “brain networks” OR “coherence” OR “rest state” OR “rest-state” OR “resting-state” OR “resting-state EEG” OR “resting-state functional connectivity” OR “resting state power” OR “resting-state power” OR “task-based” OR “task-based EEG” OR “task related” OR “task-related” OR “task related power” OR “task-related power” OR “synchronization” OR “task related synchronization” OR “task-related synchronization” OR “task related desynchronization” OR “task-related desynchronization”) AND TS=(“Motor learning” OR “Motor control” OR “Motor sequence learning” OR “Motor sequential learning” OR “Sensorimotor learning” OR “Motor imagery” OR “ Kinematics” OR “Motor training” OR “Motor practice” OR “Motor exercise” OR “ Motor expertise” OR “Motor task” OR “skill acquisition” OR “Motor cortex”) AND TS=(“older adult” OR “young adult” OR “aging”).

## 3 Results

### 3.1 Study selection criteria

The process of selecting articles for this review followed a systematic approach:

1. Initial identification: A comprehensive search was conducted to identify articles relevant to the topic. 1910 articles related to the subject were first identified.
2. Duplicate exclusion: Among the identified articles, 611 duplicates were automatically identified and excluded; 85 duplicate records were excluded by authors, resulting in 1214 unique articles.
3. Title and abstract screening: The titles and abstracts of the 1214 unique articles were meticulously assessed. As a result of this screening, 1094 were deemed not relevant to the review, and 122 were identified for potential inclusion.

#### Full text assessment

1. Accessibility confirmation: Following the title and abstract screening, accessibility to the full text of all 122 initially included articles was confirmed.
2. Comprehensive full text review: The full text of the remaining 122 was thoroughly reviewed. Adhering to the established inclusion and exclusion criteria, 42 were excluded, and 80 articles were selected for comprehensive review.

### 3.2 Data extraction

**Figure 1:**
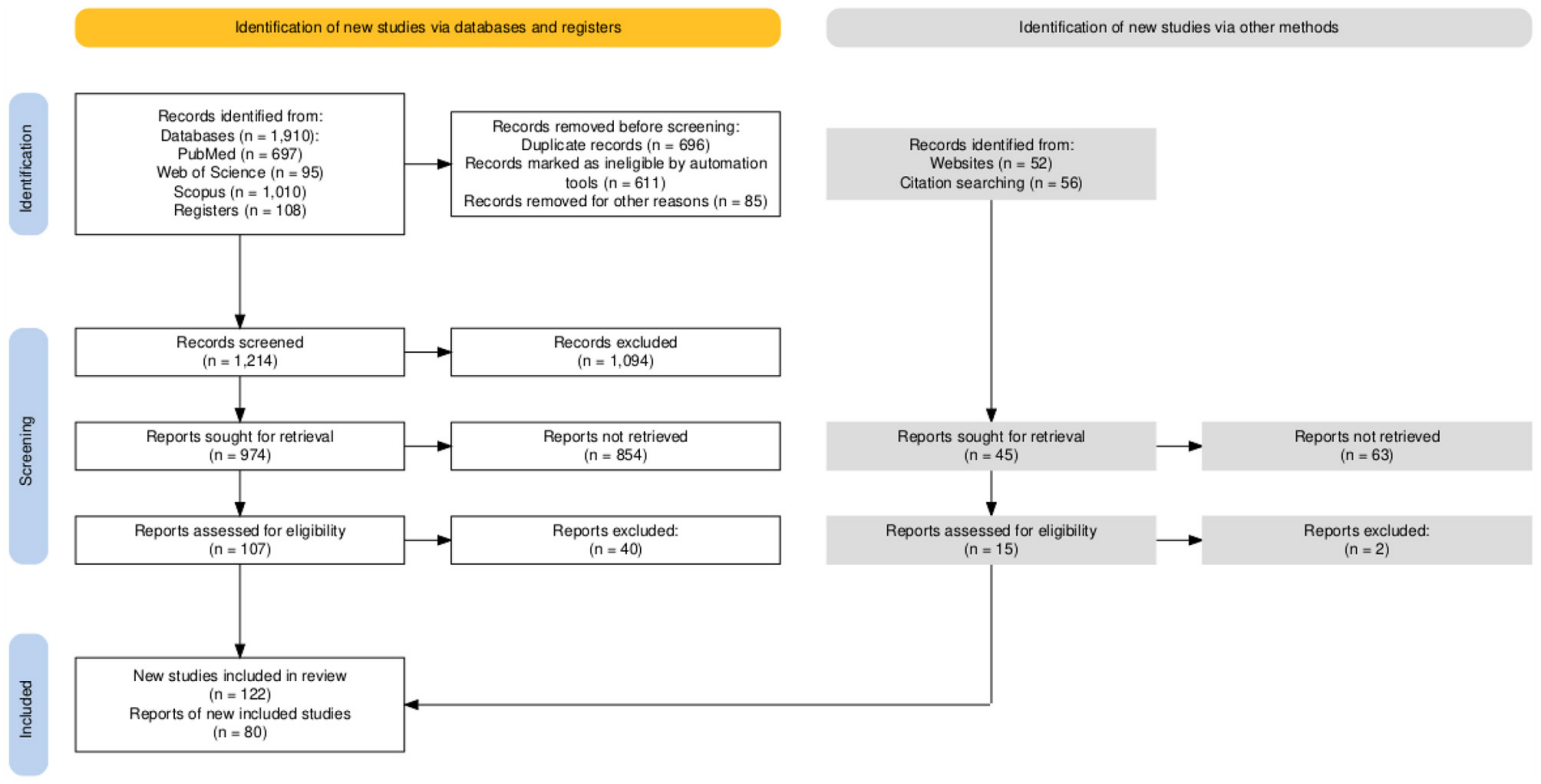
The PRISMA flow chart 2020 statement (Page et al., 2021a,b).

In the end, authors independently extracted relevant data from the studies included for this systematic review. This data encompassed various methodological and technical considerations, such as trial design, participant characteristics, experimental conditions, outcome measures, EEG parameters (e.g., frequency band, region of interest, EEG analysis method), and time point of measurement. Authors agreed about the extracted data based on the following PRISMA Flow Chart (cf., Figure 1).

### 3.3 Quality assessment

**Figure 2:**
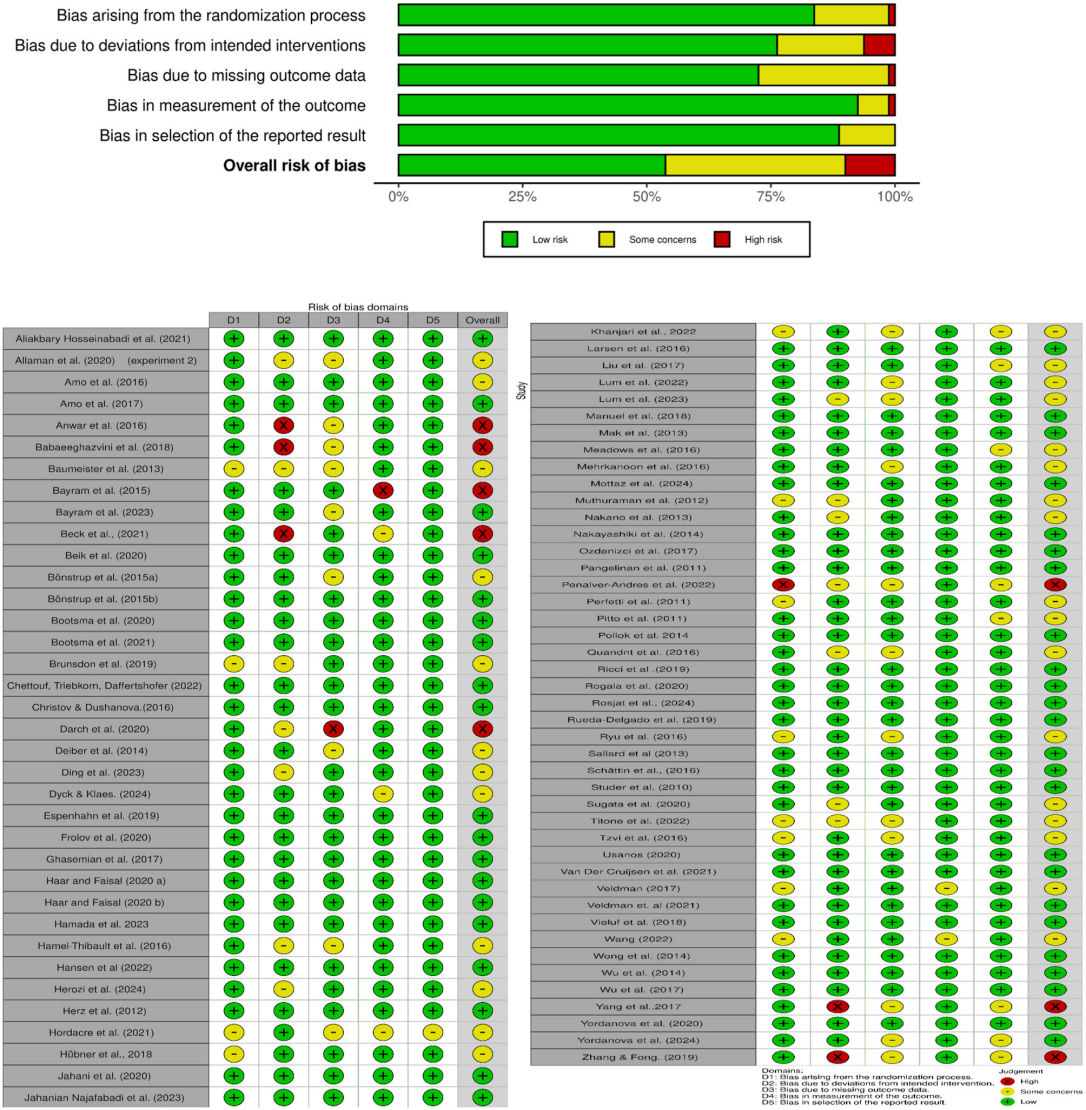
Risk of bias assessment (Higgins et al., 2016; Sterne et al., 2019).

Authors independently assessed the methodological quality of selected studies using the ROB2 tool (Higgins et al., 2016; Sterne et al., 2019). Titles of these studies, and potential abstracts, were screened independently by both authors. Titles that contained any of the exclusion criteria were excluded based on the title only. Relevant full-text articles and full texts of abstracts that were inconclusive regarding their relevance were assessed, and studies that did not correspond with the inclusion criteria were excluded. Fitting articles were also extracted from reviews relevant to the topic and full-text article references. Data regarding the studies' designs were extracted. All study designs of any methodological quality were included. Due to our objective to perform a comprehensive data collection of the various parameters and measures, we did not factor in the strength of experimental evidence provided by the studies. In addition to studies that examined the efficacy of an intervention, we included studies that explored the feasibility of tools, hypotheses regarding mechanisms of learning, recovery, and the implementation of mathematical models. In such studies, assessment of the methodological quality would yield no benefit, due to their different objectives. A narrative synthesis of the literature was performed. Figure 2 illustrates the risk of bias assessment in all categories.

### 3.4 Results

#### 3.4.1 Evidence from prior research reviewed studies

Previous research has demonstrated that brain oscillations, functional connectivity patterns involved in motor control, and EEG power spectral density serve as important biomarkers associated with motor learning outcomes. These neural indicators reflect the activity and efficiency of motor neural circuits during both task-related and resting-state conditions. As such, they offer promising tools for diagnosing pathologies within motor-related brain areas, and guiding neuro-enhancement and rehabilitation strategies aimed at enhancing the acquisition of new motor skills. However, there remains ongoing debate regarding the utility of brain oscillations as reliable biomarkers, particularly in the context of age-dependent mechanisms and their application in designing targeted rehabilitation techniques.

In the current systematic review, a total of 80 studies were included. Of these, 67 were experimental studies, and 9 were randomized controlled trials (RCTs) (Allaman et al., 2020; Beik et al., 2020; Bootsma et al., 2020; Herozi et al., 2024; Schättin et al., 2016; Studer et al., 2010; Veldman et al., 2017, 2021; Zhang and Fong, 2019) and one semirandomized (Larsen et al., 2016). Additionally, three pilot studies were included (Baumeister et al., 2013; Penalver-Andres et al., 2022; Yang et al., 2017). The earliest included study dates back to 2010. Notably, interest in the relationship between brain oscillations and motor learning surged after 2018. A temporary decline in publications was observed between 2021 and 2022, likely due to disruptions caused by the COVID-19 pandemic. A detailed overview of all included studies is presented in Table 2.

**Table 2:** Systematic review result table.

**No**.	**References**	**Participants & age range**	**Task type**	**Frequency band of interest/ROI /task-based or Resting state**	**Results**
1	Aliakbaryhosseinabadi et al. (2021)	• TN = 18 • 10 in the complex training group; *N* = 10; *F* = 5; M = 5; AR = 29.2 ± 5.1 yrs • 8 in simple training group; *N* = 8; *F* = 4; M = 4 • AR = 28.5 ± 2.6 yrs	• Simple ankle dorsiflexion and complex motor tracking tasks • The complex training group (CTG) performed a trace tracking task using their dominant foot and the simple training group (STG) executed repetitive ankle dorsiflexion in the training phase.	• Beta (13–31 Hz), and Gamma (32–8.0 Hz) bands. • Frontal, central, and parietal lobes • Task- related EEG EEG before and after the training block (pre-task, during the task, and post-task)	• Beta power decreased, and gamma power increased significantly in the frontal and central channels for the complex training group. • Theta power increased in the fronto-central channels after training. • Coherence analysis showed increased connectivity in the beta and gamma bands for complex tasks, particularly between frontal and central regions. • Indicated that brain oscillations are linked to motor skill acquisition and performance improvements. • Gamma power increased in the central and parietal lobe.
2	Allaman et al. (2020) (experiment 2)	• TN = 20 M = 7 *F* = 13 AR = (28.7 ± 5.6) 23–34 yrs	• Sequential finger tapping task • One session experiment and completed test blocks before and after the EEG block. • On four horizontally arranged buttons numbered left to right on a Chronos box, subjects were to repeat a given five-item sequence with their left hand (little finger to index)	• Alpha band & also Gamma band • M1 & dorsal premotor area • Resting- state & Task- related EEG • Eyes-closed resting state, during 10 min before the first test block • Task induced EEG during the EEG block	• Task induced alternations can be considered as the primary neural mechanism underlying behavior & performance. • Particularly spontaneous neural coupling at rest in the alpha frequency band, can be an important predictor of task performance. • Besides the primary interest band (alpha), phase coupling in Gamma frequencies influences ERD in visual areas.
3	Amo et al. (2015)	• TN = 25; M = 16; *F* = 9; AR = 18–47 yrs	• Rapid wrist bent upwards and relaxed after receiving an on-screen cue.	• Gamma band • The primary motor cortex (M1) and supplementary motor areas (SMA) • Resting- state & Task-related GBA during motor task Spontaneous gamma activity is recorded at rest	• Quantifies the variations in GBA induced by the motor task (motor GBA) in relation to basal cortical activity (basal GBA). • The mean gamma index (Iγ) was approximately 1.27, indicating a 27% increase in motor GBA over basal GBA. • No significant differences were found in EMG parameters or ERP amplitude between right and left-hand movements. • Significant correlations were observed between the EMG latency and amplitude of right and left-hand movements, as well as between the ERP amplitudes of both hands. • The gamma index (Iγ) can potentially assist in the diagnosis and rehabilitation of cortical motor area pathologies. • Increased gamma-band oscillations are associated with the planning & execution of voluntary movements, indicating a potential marker for assessing motor function in clinical populations, such as those with motor disorders.
4	Amo et al. (2017)	• TN = 16; M = 6; *F* = 10; AR = 20–47 yrs	• Quick extension of the wrist followed by the light relaxation	• Gamma band • Resting- state & Task- related EEG • Base line at rest during motor task	• Increase of the gamma band activity of both hands in movement with respect to the gamma band activity at rest. • Increased of the final gamma band activity-motor compared to the initial activity for the motor task.
5	Anwar et al. (2016)	• TN = 9; M = 4; *F* = 5; AR = 21–38 yrs	• Rhythmic & sequential finger movements of the right hand finger tapping (FT) • Simple finger sequence (SFS) • Complex finger sequence (CFS)	• The EEG-EMG coherence was used to select the peak frequency band (2–5 Hz) for subsequent source and effective connectivity analyses • Contralateral sensorimotor cortex (SMC), the contralateral premotor cortex (PMC) and the contralateral dorsolateral prefrontal cortex (DLPFC) • Resting- state & Task- related EEG	• Bi-directional effective connectivity exists between the between the PMC, and DLPFC during finger movement tasks. • Source-level EEG showed the largest GC values and significantly greater forward than backward signal flow between the ROIs • The mean Euclidean distance between the EEG and fMRI coordinates in the ROIs was within 3 mm (about 0.12 in), indicating high
6	Babaeeghazvini et al. (2018)	• TN = 48; TN (YA) = 21; M = 10; *F* = 14; AR = 21–32 yrs; TN (OA) = 17; M = 17; *F* = 7; AR= 60–74 yrs; (Final TN = 38) (final gender distribution not defined)	• A bimanual visuomotor task where participants rotated two shafts with their hands, mounted on rotating their hands while the forearms rested over ramps, which were placed on a desk.	• Beta band • Intra-hemispheric functional connectivity between dorsal premotor area (PMd) and primary motor cortex (M1) • Inter-hemispheric connections between left and right M1 • Resting- state & Task- related The results reported in the current article pertain to the behavior, obtained during the test, and resting-state EEG recordings obtained prior to the retention test, in which both young and older adults had reached a stable level of performance.	• In older adults, weaker structural connectivity between PMd and M1 in the left hemisphere was associated with stronger FC, which in turn was linked to poorer bimanual motor performance. • In younger adults, weaker structural connectivity between PMd and M1 in the right right hemisphere was associated with better performance in the simplest bimanual task. • In older adults, stronger FC between bilateral M1 regions was associated with better performance in the more complex
7	Baumeister et al. (2013)	• TN = 10 healthy athletes, M = 7; *F* = 3; AR = 19.5–22.7 yrs	• • Drop landing (DL) from a 30 cm platform • After resting, 10 athletes performed a series of DLs asked to concentrate on the landing preparation for 10 s before an auditory signal required them to drop land from a 30 cm platform.	• Theta band (4.75–6.75 Hz) • Alpha-1 band (7.0–9.5 Hz) • Alpha-2 band (9.75–12.5 Hz) • Frontal brain areas (especially mid-frontal electrodes F3, Fz, F4) • Central areas (C3, Cz, C4) • Parietal areas (P3, Pz, P4) • Resting- state EEG (during the 10-s preparation period before the drop landing, and after the fatigue fatigue protocol)	• Increased frontal Theta power during the preparation period compared to rest, indicating higher attentional control • Increased Parietal Alpha-2 powers after the fatigue protocol, suggesting a deactivation in the somatosensory cortex • No significant changes were observed in Theta and Alpha-1 power values after the fatigue protocol • Predictive sensorimotor control during landing preparation involves increased frontal Theta power for attentional control and that fatigue induces increased parietal Alpha-2 power, possibly reflecting somatosensory cortical inhibition, with stable motor execution patterns
8	Bayram et al. (2015)	• TN (OA) = 28; *F* = 20; M = 8; AR = 74.96 ± 1.32 yrs • TN (YA) = 20; *F* = 10; M = 10; AR = 22.60 ± 0.90 years; Overall AR = 21.7–76.3 yrs	21.7–76.3 yrs	• Maximal Voluntary Contraction (MVC) • Submaximal Elbow Flexion (EF) at 20%, 50%, and 80% MVC	• Beta band (15–35 Hz) • EEG Electrode Clusters: Right sensorimotor area (C4) and central area (Cz) • Right frontal-parietal areas in young subjects and parietal region in older subjects • Task- related EEG • Lower Corticomuscular coherence (CMC) in older adults compared to younger adults at all force levels. • A proportional relationship between CMC & force in both groups, with a strong positive correlation between CMC and MVC force. • A significant weakening of CMC during voluntary motor actions in older adults compared to younger individuals. • Higher CMC for the BB and BR muscles compared to the TB muscle, which acted as an antagonist • Decline in CMC with aging may be a critical factor in the observed decrease in motor performance & muscle strength among older adults.
9	Bayram et al. (2023)	• TN (YA) = 20; *F* = 10; M = 10; AR = 21.73–23.47 yrs • TN (OA) = 28; *F* = 20; M = 8; AR = 73.42–76.16 yrs	• Voluntary movement task • voluntary elbow flexion (EF)	• Beta band • Primary motor cortex, primary sensorimotor area, contralateral to the left elbow flexors. • Task-related EEG	• Beta-band relative ESP in elderly did not significantly decrease with increasing EF force values • The elderly group exhibited significantly lower EF strength compared to the young group, likely due to reduced neural drive to the muscle group • The total ESP did not significantly differ between the mal voluntary contraction (MVC), groups except at 80% maxi where it was • The absolute ESP in the beta and low-gamma bands was significantly lower in the elderly at 80% MVC • The relative ESP in the beta band was significantly higher in the elderly at 20% and 50% MVC showed a different relationship between absolute ESP and force across all EEG bands, and a different relationship between relative ESP and EF force except in the theta band
10	Beck et al. (2021)	• 114 individuals, with data from 109 EEG-EMG files for the dominant hand & 111 for the non-dominant hand analyzed • Children aged 8–10 years, adolescents aged 12–14 years, & adults aged 20–30 years	• Tonic force-tracing task using a precision grip	• Beta band (15–30 Hz) • Cortical Sources: Coherent activity was localized in the pre-central & post-central gyrus of the contralateral sensorimotor cortex, extending into the superior parietal lobule and the middle frontal gyrus	• Greater levels of beta-band corticomuscular coherence were observed in adults compared to children • Adults showed greater motor precision, and less variability compared to younger age groups • Higher coherence levels were found in the non-dominant hand compared to the dominant hand, although the effect size was small
11	Beik et al. (2020)	• TN = 60 OA • M = 60 • AR = 65–75 yrs	• Novel hand actions pressing keys • The task was sequentially depressing the 2, 5, 6, 9 keys with the dominant (right) hand in Absolute Timing Goals (ATGs).	• Alpha (8–12 Hz) & Beta band (14–30 Hz) • Sensorimotor & central electrodes frontal (Fp1, Fp2, F3, F4), central (C3, C4), & parietal (P3, P4) cortices • Percentage change in power for each condition relative to the pre-/post-resting EEG • Task-related EEG • EEG signals were continuously collected during the acquisition phase & delayed retention	• Greater alpha & beta desynchronization during hand hand action observation compared to static hand observation • Greater power over the occipital electrodes compared to the central electrodes • Execution training group exhibiting significantly greater alpha power overall than the observation training group • Alpha power activity to the hand action significantly increased from pre- to post-training in the execution training group, but did not change in the observation training group • No significant difference in pre- to post-training for Beta power
12	Bönstrup et al. (2015a)	• TN = 30 YA/OA • M = 13 • *F* = 17 • YA. AR = 25 ± 2.6 years • OA. AR = 70 ± 3.2 years per group one participant had to be excluded from data analysis due to technical problem but final gender distribution not defined	• a complex finger-tapping sequence	• Upper Alpha band (12–14 Hz) • Resting- state & Task- related • EEG Pre task • Prefrontal cortex & parietal areas	• Young participants showed a significant increase in alpha power at sensorimotor cortices during inhibition of the learned motor sequence, reflecting active motor inhibition. • Elderly participants showed significantly less or absent alpha power increase during inhibition, indicating impaired cortical inhibitory control. • Both groups showed alpha power decrease during execution of the motor sequence, but elderly showed a smaller inhibitory alpha power increase during withholding the movement. • Overnight consolidation (24 h after learning) enhanced alpha power increase during inhibition in young participants but not significantly in elderly, suggesting reduced neuroplasticity or delayed inhibitory control strengthening with age.
13	Bönstrup et al. (2015b)	• TN = 14 • M = 7 • *F* = 7 • AR = 27.7 ± 2.8 yrs	• Repetitive simple, near-isometric whole-hand grips • Visually instructed isometric hand grips	• Alpha (9–13 Hz), Beta (14–30 Hz) bands • M1, ventral premotor cortex (PMv) and SMA bilaterally • EEG recorded during motor task with rest period	• A dominant coupling within the β-band (13–30 Hz) between contralateral M1 & SMA during isometric contraction of the forearm • Task-related spectral dynamics in the alpha-to-beta frequency range, with bilateral M1 showing task-related power changes • Grip-related increases in facilitatory coupling between SMA & M1 in the contralateral hemisphere
14	Bootsma et al. (2020)	• TN (OA) = 36 • M = 20 • *F* = 16 • AR = 70.4 ± 4.1 yrs (65–86 yrs)	• Continuous tracing task • Mirror star-tracing	• Alpha & beta bands • Frontal cortex, motor cortex, parietal cortex • Resting- state & Task- related EEG	• Due to frontal channels improvement in cup stacking performance are related to α & β power reductions•Due to Central channels improvement in cup stacking performance is related to α & β power reductions • Due to Parietal channels improvement in cup stacking performance is related to β power reductions
15	Bootsma et al. (2021)	• TN (YA) = 36 • M = 16 • *F* = 20 • AR = 19–24 yrs • TN (OA) = 36 • M = 20 • *F* = 16 • AR = 65–86 yrs	• Mirror star tracing task at one of three difficulty levels. Unimanual reaching movements toward the appearing visual targets • On day 1, participants practiced the visuomotor task at one of three difficulty levels. Before (baseline), immediately after (Post) & 24 h after (Retention) practice, motor performance, & EEG data were acquired. • The mirror star-tracing task. Participants were asked to trace the outline of a symmetrical five-point star as quickly and accurately as possible, only being allowed to look at the star and their moving hand through a mirror. • The combinations of electrodes used for the regions of interest in the EEG analysis	• Alpha & beta band • Resting- state & task- related • Frontal cortex, motor cortex, parietal cortex	• Hand selection & reach reaction times strongly depend upon the instantaneous phase of delta at the moment of target onset. • This effect was maximal over contralateral motor regions & occurred in the absence of pre-stimulus alpha & beta-band amplitude modulations. • The excitability of motor regions acts as a modulatory factor for hand choice during reaching.
16	Brunsdon et al. (2019)	Initial TN: 67 • Execution training group • TN = 60 • M = 38 • *F* = 22 • AR = 18–30 yrs • Observation: training group • TN = 60 • M = 40 • *F* = 20 • AR = 18–39 yrs	• Observing and performing unfamiliar hand actions AND half completed completed observation-only training • Execution training group: Participants physically executed hand actions while watching videos. • Observation training group: Participants only watched videos of hand of hand actions being performed. • Pre- and post-training EEG tasks involved observing short video clips showing unfamiliar hand actions or a static hand image.	• Alpha (8–13 Hz) & beta band (13–35 Hz) • EEG recorded during pre- & post-training tasks only • Central electrodes over sensorimotor cortex (C3, Cz, C4) Occipital electrodes (O1, Oz, O2) • Task-related & Resting state EEG (used to calculate desynchronization/synchronization relative to resting power)	• Both alpha and beta desynchronization in the sensorimotor cortex were significantly greater during hand action observation than during static hand observation. • After training, only the execution training group showed increased alpha desynchronization during hand action observation; the observation group showed no change. • Short-term physical rehearsal enhances sensorimotor cortex activation in alpha and beta bands during action observation, based on changes from resting state EEG recorded before and after training.
17	Chettouf et al. (2022)	• TN = 20 YA • *F* = 13 • M = 7 • AR = 20–25 • TN = 20 OA • *F* = 14 • M = 6 • AR = 59–70 Final TN = 27 (13 younger, 14 older) gender not mentioned	• Unimanual motor task: Squeeze in an air-filled rubber bulb with their right hand in a 4:3 frequency ratio to an external cue to match the visual feedback where two discs appear to rotate in sync at a 1:1 frequency ratio.	• Beta Band (15–30) Hz providing supplementary results for the alpha band (8–14 Hz) • Primary motor cortex (M1), especially the contralateral (left) M1, Bilateral premotor cortex (PM1) • Task-related & Resting state EEG	• Motor-event related β-band power was lateralized contralaterally in both groups, with stronger lateralization in younger adults. Older adults showed a significantly lower mean β-power during motor execution in bilateral premotor areas. • Combined EEG-fMRI displayed Positive correlations between β-amplitude and BOLD in primary motor cortex and negative premotor cortex were observed in both groups. Older adults showed a negative correlation in a small left M1 region. No significant group differences in combined EEG-fMRI correlations. • Decreased β-power in premotor areas in older adults, suggesting diminished intra-hemispheric inhibition may lead to more interhemispheric crosstalk and poorer motor performance with age.
18	Christov and Dushanova (2016)	• TN = 48 • TN YA = 24 • *F* = 11 • M = 13 • AR = 25–31 yrs • TN OA = 24 • *F* = 11 • M = 13 • AR = 55–60 yrs	• Auditory discrimination task • The effect of aging on beta (β1: beta (β1: 12.5–20; β2: 20.5–30 Hz) & gamma (γ1: 30.5–49; γ2: 52–69 Hz) band components of ERP was studied in an auditory discrimination task (low-frequency & high-frequency tone) at frontal, central, parietal and occipital cortical locations at short latency (post-stimulus interval 0–250 ms; putative sensory processing) & long latency (250–600 ms; putative cognitive) periods.	• Beta and gamma bands beta (β1: 12.5–20; β2: 20.5–30 Hz) and gamma (γ1: 30.5–49; • Frontal, central, parietal and occipital cortical locations at short latency (post-stimulus interval 0–250 ms; putative sensory processing) and long latency (250–600 ms; putative cognitive) periods. • Task-related EEG	• Beta1 component of the short latency period of ERBRs was less affected by age. The beta1 activity of the long latency period was reduced by age and more widespread than in the short latency The aged difference in beta1 component spread into fronto-parietal regions and was more expressed after high-frequency after high-frequency than after low tone stimulation. • Beta2 and gamma amplitudes were higher with processing. Reducing regional-process specificity with progressing age characterized tone-dependent • Beta2 changes during short latency (sensory), but not during long latency (cognitive) processing. • Late latency (cognitive) beta2 and gamma activity diminished with age, except for the frontal high tone responses. • With increasing age, gamma2 activity was more expressed over the frontal brain areas in high tone discrimination.
19	Darch et al. (2020)	• TN = 11 adult • *F* = 4 • M =7 • AR = 21–31 yrs	• Human task: joystick visuomotor adaptation task Cats task: prism visuomotor adaptation task	• Beta band • Task-related EEG • Primary motor cortex and the sensorimotor cortex • In animal studies, specific sites within the motor cortex are targeted for local field potential (LFP) (LFP) recordings	• Pre-movement beta frequency oscillations in the motor cortex predict motor adaptation drive. • Beta (15–25 Hz) power recorded over the motor cortex was significantly reduced in amplitude during early adaptation trials compared to baseline, late adaptation, or aftereffect trials. across humans and cats, indicating a generalizable phenomenon related to motor cortical activity during early stages of adaptation.
20	Deiber et al. (2014)	• TN = 62 • TN (YA) = 30 • *F* = 19 • M = 11 • AR = 24.4 ± 2.5 years • TN (OA) = 32 • *F* = 18 • M = 14 • AR = 68.7 ± 5.7 years	• Three delayed motor tasks • Participants performed unilateral key presses with the left or right index finger under three different pre-cued conditions: (1) Full, where S1 provided complete advance information about response side; (2) Free, where S1 invited the participant to choose the side of response and (3) None, where S1 was uninformative on the response side.	• Mu (9–12 Hz) & low beta (15–20 Hz) • Task-related EEG • Focused on the responsiveness of the sensorimotor region in preparatory processes for internally vs. externally guided responses, as indexed by the MRAA. • Focused on the lateral premotor network and the mediofrontal network in	• Older adults showed reduced mu lateralization and increased beta lateralization compared to younger adults, especially internally guided movements, which is associated with slower reaction times in older adults. • Older adults exhibited slower reaction times compared to younger adults across all conditions (Full, Free, None) • The interaction between age group & condition was significant, with older adults showing longer RTs in the Free condition compared to the Full condition, a pattern not observed in younger adults • Both age groups maintained high accuracy levels (>98%) across conditions. Older adults performed worse than younger adults in the Full condition but better in the None condition • Older adults showed significant beta MRAA in both Full and Free conditions, with larger & longer-lasting beta MRAA in the Full condition compared to younger adults
21	Ding et al. (2023)	• TN = 30 • M= 8 • *F* = 22 • AR = 21.6 + 1.5 years	• The Stop Signal Reaction time Task (SSRT) used to assess the ability of motor inhibitory control • On “Go” trials, a black arrow was presented on the screen, & participants were instructed to press the left-arrow key for a leftward pointing arrow with the left index finger, and to press the right-arrow key for a rightward pointing arrow with the right index finger. • On “Nogo” trials, a red arrow was presented on the screen, and participants were instructed not to press any key. On “Stop” trials, a “Stop” signal (red arrow) would occur after the “Go” signal (i.e., the black arrow turned red after a delay). Participants were asked to stop their initial response when the “Stop” signal occurred. Participants were instructed to respond as quickly & accurately as possible to black arrows, and not to delay their response to wait in case the “Stop” signal occurred	• Beta • left and right motor cortex (MC), right somatosensory cortex (SC), and right inferior frontal cortex (IFC) • Task-related & Resting state cortical beta oscillations • EEG was performed after the completion of SST. The participants were seated comfortably in a sound-shielded, dimly lit room for resting-state EEG recording (13 min): eyes closed (6 min), followed by 1 min with eyes open, and 6 min with eyes closed (6 min).	• Significant positive correlations were found between SSRT and beta band power in the left and right motor cortex (MC), right somatosensory cortex (SC), and right inferior frontal cortex (IFC). This indicates that individuals with poorer response inhibition had greater beta power in these regions • Positive correlations were observed between SSRT & beta band coherence between pairs of electrodes in bilateral MC, SC, and IFC. This suggests that higher functional connectivity in these regions is associated with poorer response inhibition.
22	Dyck and Klaes (2024)	• TN = 25 • M = 7 • AR = 18–26 yrs (final TN = 19 gender distribution not defined)	• A modified version of the Serial Reaction Time Task (SRTT) • Instead of providing spatial cues, color stimuli have been employed to guide the motor responses in an adapted SRTT	• Beta band • Task-related EEG • Central regions • Throughout the task, including the planning & execution phases of each stimulus presentation.	• Stronger beta suppression was observed early in explicit learning, followed by an increase in beta power over time, indicating cognitive involvement in motor sequence learning • Faster RTs and higher accuracy rates are rates are associated with explicit learning conditions compared to implicit and random conditions • Changes in beta ERSPs correlate with behavioral performance, suggesting beta ERD as a potential biomarker for motor sequence learning
23	Espenhahn et al. (2019)	• TN (OA) = 20 (final TN =38 gender distribution not defined) • M = 7 • *F* = 12 • AR = 62–77 • TN (YA) = 20 • M = 8 • *F* = 11 • AR = 18–30	• a novel wrist flexion/extension tracking task & subsequently retested at two different time points (45–60 min and 24 h after initial training). Scalp EEG was recorded during a separate simple motor task before each training & retest session	• Beta (15–30 Hz) • Sensorimotor cortex, left superior parietal lobe, inferior parietal lobe & left precentral gyrus • Task-related EEG	• Motor performance during training was a robust predictor of subsequent performance, but movement-related beta activity explained more variance in individual motor performance 45–60 min after training, but not 24 h later. • Cortical beta oscillations in the sensorimotor cortex are associated with short-term motor performance following visuomotor learning and may serve as biomarkers for individual differences in motor learning capacity.
24	Frolov et al. (2020)	• TN (OA) = 10 • M = 4 • *F* = 6 • AR = 55–72 • TN (YA) = 10 • M = 7 • *F* = 3 • AR = 19–33	• A fine motor task involves squeezing one of their hands into a fist after an audio signal and holding it until a second signal. They performed this task 30 times with each hand, for a total of 60 repetitions	• Mu, Beta & Theta bands • Task-related EEG	• Older adults showed significantly slower motor brain response time (MBRT) compared to young adults, particularly in mu (8–14 Hz) and beta (15–30 Hz) frequency bands. • In young adults, motor cortex activation was faster during dominant hand movements, while in elderly adults, motor activation was equally slow for both hands, indicating a loss of dominant hand advantage (approach • Elderly adults exhibited increased theta-band (4–8 Hz) activation broadly in frontal, central, and parietal EEG sensors during the motor initiation period, unlike young adults who showed less pronounced theta activity. • Functional connectivity analysis revealed stronger midline theta connectivity in young adults, associated with motor working memory and perceptual-motor facility. • In contrast, elderly adults had increased theta-band connectivity involving central, temporal, and frontal regions, suggesting reliance on more sensorimotor integration mechanisms.
25	Ghasemian et al. (2017)	• TN = 17 • M = all male • AR = 18–22 yrs	• pursuit tracking motor skill task	• Theta. alpha & beta band • Frontal, central, parietal electrodes (contralateral motor cortex) • Task- related EEG • On the first day, participants initially performed the task as a pretest. Then, they practiced the task within three blocks of five trials. Each trial lasted 60 seconds; at the end of the day, they participated in a posttest like the pretest (n¼3 trials). Participants then practiced motor skills every other day for 5 days.	• Theta (θ) power increased at frontal site Fz on the last training day compared to the first day. • Alpha (α) power increased at central site Cz on the last day compared to the first. • Alpha coherence between Fz-T3 and Fz-Cz decreased over the course of training. • Beta (β) coherence between Fz-Cz was significantly reduced from pre- to post-training.
26	Haar and Faisal (2020a)	• TN = 30 • M = 18 • *F* = 12 • AR = 24 ± 3 yrs	• A real-world pool table billiards task • Brain activity was recorded using wireless EEG, & ball movements were tracked with a high-speed camera • Participants repeated trials of a pool shot task, divided into 6 sets of 50 trials each, with short breaks in between	• Beta band • Left motor cortex., right motor cortex • Task- related EEG	• Two distinct groups were identified based on PMBR dynamics: PMBR Increasers & PMBR decreases • PMBR Increasers showed an increase in PMBR over learning, while PMBR Decreases showed a decrease • PMBR increasers likely used error-based adaptation, while PMBR Decreases likely used reward-based reinforcement learning • PMBR decreases exhibited higher initial variability & a steeper decrease in variability over trials, indicating more exploration early in learning
27	Haar and Faisal (2020b)	• TN = 30 • M = 18 • *F* = 12 • AR = 24 ± 3 ys	• Shooting a target ball toward a pocket on a billiards table	• Beta band • Subcortical regions, cerebellum, basal ganglia • Task- related EEG • EEG was recorded during the task, specifically throughout the entire learning process as subjects performed repeated trials of a billiards task	• Two groups of subjects were identified based on PMBR dynamics: dynamics: Increasers & PMBR decreases • PMBR increasers showed an increase in PMBR over learning, negatively correlated with decreasing directional errors, suggesting error-based adaptation • PMBR Decreases showed a decrease in PMBR over learning, positively correlated with decreasing directional errors, suggesting reward-based learning • PMBR decreases showing more exploration & a steeper decrease in
28	Hamada et al. (2023)	• TN = 15 • M = 10 • *F* = 5 • AR = 22.8 ± 3.0 yrs	• Ball rotation task clockwise (CW) & counterclockwise (CCW) rotations	• alpha (8–13 Hz), beta (13–30 Hz), Low gamma (30–50 Hz), High gamma (50–80 Hz) bands • Task-related EEG • Regions were identified based on significant differences in EEG power spectra • Right temporal region (fusiform gyrus) left parietal area (CP3 and P3): corresponding to the superior parietal lobule frontal region (right premotor area & supplemental motor area), right temporal region: right frontal region. • Left lateral parietal region	• The number of rotations in the CW task increased significantly from 11.6 ± 1.9 in the pre-test to 25.5 ± 2.6 in the post-test. No significant changes were observed in the CCW task • Excess of low & high gamma band were observed in the right frontal, left parietal, & right temporal regions during the post-test. • Positive correlations between the number of rotations & EEG power values in the right frontal & right temporal regions • No significant increase in EMG activity was observed, suggesting that changes in motor patterns were more important than increases in muscle activity • Neuromodulation interventions targeting gamma band oscillations in specific brain regions could enhance motor learning efficiency
29	Hamel-Thibault et al. (2016)	• TN = 19 • M = 11 • *F* = 8 • AR = 21–34 yrs	• Simple motor task to make a transition between two force levels	• Delta (2–4 Hz), alpha (8–12 Hz), beta bands (15–30 Hz) • Task-related EEG • Contralateral motor regions	• Hand selection and reach reaction times (RTs) were strongly dependent strongly dependent on the instantaneous phase of delta oscillations at target onset. • This dependency was maximal over contralateral motor cortical regions. • Delta phase in the motor cortex contralateral to the selected hand was related both to which hand was chosen and how fast the reach was executed. • There were no significant prestimulus modulations in alpha (8–12 Hz) or beta (15–30 Hz) band amplitudes related to
30	Hansen et al. (2022)	• Total TN = 22 • Training group • TN = 12 • M = 9 • *F* = 3 • Control group • TN = 10 • M = 8 • *F* = 2 • Overall AR = 20.81–24.91 yrs	• The study on motor learning improvement focused on a cup stacking task, which is a highly complex bimanual task that relies heavily on visuo-motor coordination and motor planning • The task type involved participants performing a series of cup stacking trials, where they had to “up-stack“ & “down-stack“ cups as quickly as possible	• alpha (8–13 Hz), beta (13–30 Hz), and gamma bands (31–50 Hz) • EEG recordings were conducted during the cup stacking task, where participants performed five attempts to complete the task while surface EEG was recorded throughout the entire attempt • Additionally, a 30-s resting-state EEG recording was conducted prior to the cup stacking protocols on each test day • The resting-state EEG data was used to normalize the cup-stacking data across different participants & test days	• High- & low-motor learning groups exhibited different EEG activities when learning a new motor skill • Not only during action execution but also during the observation of & preparation for • Participants in the training group showed significant improvements in their cup stacking performance, with a considerable reduction in the time to complete the task from Pre-test to Post-test. This improvement was retained up to seven days after the training session • Training-related changes were observed in brain areas associated with motor control, somatosensory integration, & motor planning. Specifically, there were reductions in alpha & beta power in the central, parietal, and frontal EEG channels immediately after the training session • Retention of Motor Patterns: The training group retained their new motor patterns, group continued to improve with each trial • EEG Power Changes: The reductions in alpha & beta power in the frontal, central, and parietal areas suggest that these regions are involved in the early stages of motor learning & that their activity decreases as the task becomes more automated • Task Complexity and Cognitive Engagement: The cognitive complexity of the cup stacking task likely required engagement of additional neuronal circuits, as indicated by the reduced alpha power in the frontal area
31	Herozi et al. (2024)	• TN = 26 • M = 26 • *F* = 0 • TN (OA) = 12 • AR = 55–70 yrs • TN (YA) = 14 • AR = 20–30 yrs	• Digital mirror-tracing task	• Alpha band • Resting state & Task-related EEG • Frontocentral and bilateral inferior frontal cortical areas • Impact of binaural acoustic beat (BAB) training on motor learning in young and older adult individuals	• Alpha band activity may improve neural dynamics in young & older adults, potentially enhancing motor planning & aptitude. • Further research is needed on imaging & neuromodulation modalities evaluation of concurrent BAB and minimally invasive brain stimulation. • Theta absolute power was observed in young groups • Alpha: the use of alpha binaural beats (BAB) to enhance motor learning & performance • Involvement of Low Beta (12–16 Hz) in motor performance and learning involvement of high beta oscillations in older adults • Gamma (25–45 Hz) in the left frontocentral cortical zone in
32	Herz et al. (2011)	• TN = 13 • M = 7 • *F* = 6 • AR = 18–26 yrs	• Two unimanual motor tasks: fast self-paced extension-flexion movements with the right index finger (referred to as FAST), an isometric contraction of the right forearm (referred to as ISO).	• θ-band (4–7 Hz), β-band (13–30 Hz), γ-band (31–48 Hz) • Alpha, Beta, Gamma bands • Task-related EEG • left primary motor cortex (M1), the lateral premotor cortex (lPM), & the supplementary motor area (SMA) in the left hemisphere.	• The Bayesian model selection favored a model that accounted for both within- & cross-frequency coupling in both extrinsic & intrinsic connections. • Strong coupling within the γ-band and between the θ- and γ-bands was observed during the FAST condition, primarily in connections from lPM to SMA and M1. • ISO condition was characterized by significant coupling coupling within the β-band, mainly in the connection between M1 & SMA. • The study revealed task-specific modulation of inter-regional oscillatory coupling within the core motor network, suggesting that the frequency of oscillations may determine which connections of a network are activated.
33	Hordacre et al. (2021)	• TN = 18 • M = 8 • *F* = 10 • AR = 18–43 yrs	• The motor task involved recording motor evoked potentials (MEPs) from the right first dorsal interosseous (FDI) muscle following TMS applied to the left M1	• High beta band 20–30 Hz) & Low beta band (14–19 Hz) • Frontocentral region, contralateral parietal region, & contralateral occipital region • Rest EEG, prior to any brain stimulation	• Resting-state functional connectivity in the high beta frequency band (approximately 20–30 Hz) was the strongest predictor of response to cTBS. • Resting-state EEG connectivity may thus serve as a biomarker for responsiveness to brain stimulation interventions.
34	Hübner et al. (2018)	• TN = 38 OA • M = 16 • *F* = 22 • AR = 65–74 yrs	• A precision grip force modulation (FM) task	• Beta frequency band (13–30 Hz) • Frontal cortex (specifically, contralateral to the performing hand) • Sensorimotor cortical areas (M1, S1), bilaterally The study particularly analyzed electrodes over the frontal, central, and centro-parietal region • Resting state, task related EEG During the FM task and at rest before and after the sessions.	• The acute exercise group showed greater immediate improvement in fine motor performance after exercise compared to the resting control group, with a marginal significance. • Exercise enhanced consolidation of short-term and long-term motor memory. EEG beta activity (task related power decreases) was stronger immediately after exercise over contralateral frontal cortical areas, possibly indicating enhanced cortical activation and compensatory processes. • No significant long-term effects on EEG beta power at rest or on motor memory consolidation were confirmed, though some tendencies suggested benefits might depend on exercise load.
35	Jahani et al. (2020)	• TN = 24 • M = 16 • *F* = 8 • AR = 20–32 yrs (final TN = 23 gender distribution not defined)	• Ballistic movements with no online corrections. • A motor adaptation task in which a visual rotation was introduced in short series of trials separated by unperturbed trials. • Participants were instructed to “shoot,” without stopping, one of three possible visual targets	• Beta band • Task-related EEG • Sensorimotor regions, medial motor areas	• Beta-band activity in lateral central regions was significantly modulated during the four period of tasks, which correlates with implicit adaptive triggered by movement-execution errors.
36	Jahanian Najafabadi et al. (2023)	• TN (OA) = 41, • Final = 31 • M = 22 • *F* = 19 • AR = 68.92, SD: 4.49 • TN (YA) = 37, final 26 • M= 15 • *F* = 19 • AR = 23.64, SD: 7.07	• A virtual tool-use training in AR, visual feedback with/ without vibro-tactile feedback	• Beta band, • Resting state, task related EEG • Sensorimotor, frontal, central, parietal, & occipital areas	• Greater beta relative power predicts stronger Practice effect in younger adults • Lower Beta relative power predicts stronger practice effect in older adults
37	Khanjari et al. (2023)	• 14 non-athlete students • M = all male • 7 participants with right-hand dominance • 7 participants with left-hand (non-dominant hand) • AR = 21–25 yrs	• Dart skill learning • Dart throwing using both dominant and non-dominant hands.	• Beta band (13–30 Hz) • Frontal region (AF3, AF4) Central region (CP5, CP6) Parietal-occipital region (PO3, PO4) • Task-related EEG • EEG signals were recorded during the dart throwing skill in three phases: pre-test (before training), during acquisition, and retention (after training).	• During dart skill learning, EEG Beta activity showed significant changes in frontal, central, and parietal-occipital regions. • Increased activity (energy) was observed in frontal (AF3, AF4) and parietal-occipital (PO3, PO4) regions, reflecting cortical engagement in learning. • There was a decrease in activity in some central regions (CP5, CP6) over time. Both dominant and non-dominant hand groups showed learning-related changes, but the EEG patterns differed between these groups. • Despite improvements in dart accuracy, cortical activity remained relatively stable, supporting the idea that neural reorganization underpins motor learning • The cortical maps suggest that separate motor programs likely control dominant and non-dominant hands, aligning with the proficiency model.
38	Larsen et al. (2016)	• TN = 16 • *F* = all female • AR = 21–29 yrs	• Precision pinch movement tablet-based motor practice game	• Beta band (15–30 Hz) • Right primary motor cortex (electrode C4) • Task-related EEG	• Tablet-based motor practice involving fast and precise pinch movements with thumb and index fingers of the non-dominant hand improved motor performance—the number of pinched crabs increased over three 10-min blocks of practice. • Tablet practice improved manual dexterity; increased corticomuscular and intermuscular coherence in the beta band, indicating increased corticospinal drive • Corticomuscular coherence (CM coherence) between EEG from primary motor cortex and EMG from finger muscles (abductor pollicis brevis [APB] and first dorsal interosseous [FDI]) increased significantly after tablet-based practice in the beta frequency band, indicating enhanced corticospinal drive to the spinal motoneurons controlling finger muscles. • Intermuscular coherence (IM coherence) between EMG of APB and FDI also increased in the beta band after practice.
39	Liu et al. (2017)	• TN (OA) = 24 • TN (YA) = 18 • AR (OA) = 60–78 yrs	• Finger tapping task	• Beta, alpha bands • Task-related EEG	• Phase locking in the δ-θ frequency band, which is band, which is associated with movement execution, is not affected by age. • Post-movement beta amplitude is diminished in older subjects & is related to age-related deficits in motor accuracy. • The post-movement beta amplitude in the medial prefrontal cortex (mPFC) positively correlates with the accuracy of motor performance in older subjects.
40	Lum et al. (2023a)	• TN = 50 • M = 17 • *F* = 33 • AR = 19–37 yrs	• Serial reaction time task; unknowingly repeat a sequence of finger movements in response to a visual stimulus	• Theta, beta, & alpha bands • Task related EEG • The primary motor cortex, the supplementary motor area, & the prefrontal cortex	• Changes in theta power are significantly associated with the learning of implicit motor sequences. • Increased theta power correlates with improved performance on the SRT task, suggesting that theta oscillations play a critical role a critical role neural processes involved in implicit learning. • Older adults may exhibit deficits in implicit motor sequence learning compared to younger individuals. • Relationship between increased theta power and enhanced performance in the SRT task, reinforcing the notion that brain oscillations are integral to
41	Lum et al. (2023b)	• TN = 85 • M = 33 • *F* = 52 • AR = 18.3 & 59.2 years	• Serial reaction time task; unknowingly repeat a sequence of finger movements in response to a visual stimulus	• Theta, alpha & beta bands • Resting-state EEG data were acquired recorded before the task to examine correlations between EEG spectral spectral power and implicit learning	• Lower levels of theta power during the SRT task may promote implicit sequence learning by disengaging explicit learning and memory mechanisms • Lower levels of theta was associated with implicit sequence learning on the SRT task implicit sequence learning would be negatively correlated with resting state beta and theta power and positively correlated with resting state alpha power.
42	Manuel et al. (2018)	• Exp Group TN = 12 • M = 7 • *F* = 5 • AR = aged 22 ± 4 • Ctrl Group TN = 12 • M = 7 • *F* = 5 • AR = aged 23 ± 5	• Mirror drawing task • Exp group performed a computerized classic mirror-drawing task • Control group performed a similar task but with concordant direction of cursor movement as a measure of motor execution.	• Alpha, beta bands • Frontal and central electrodes • ERD, ERS • Resting state/Task-related EEG	• No association between parietal or motor functional connectivity networks during the first 20 min after training and offline consolidation. These results indicate that the left parietal resting-state network engaged before training plays a specific role in preparing the brain for the upcoming tasks rather than contributing to the consolidation of training gains • Alpha-band wholebrain FC is primarily implicated in providing optimal neural resources before a task. This clarifies previous evidence for between alpha-band FC and behavioral performance
43	Mak et al. (2013)	• TN = 10 M = 7 *F* = 3 AR = 26 ± 3 yrs	• Computerized visual-motor task similar to mirror drawing • Trace a heptagon on a computer screen with a mouse, while the screen/mouse movement was reversed left-right, mimicking the classic mirror-drawing paradigm	• Delta (1–4 Hz) • Gamma (36–44 Hz) • Only the frontal region (forehead) was examined, given the single-channel headset design • EEG was recorded during the motor task	• As participants became more familiar with the task with the task through repetition: • EEG power in all bands generally decreased, especially in delta and gamma bands. • Delta band power (frontal EEG) was significantly negatively correlated with both task accuracy and the normalized familiarity index for the entire trial. • Gamma band power (frontal EEG) was significantly and negatively correlated with task familiarity, suggesting its potential as a marker for early learning progress. • The study demonstrates the possibility of monitoring motor learning progress using • consumer-grade, single-channel frontal EEG devices • Decreases in Delta and Gamma power indices correlated with increased task familiarity and performance.
44	Meadows et al. (2016)	• TN = 20 M = 15 *F* = 5 AR = 22.3 ± 3.6 yrs	• 4 blocks of 42 trials of a reaction time task by squeezing a hand dynamometer in dynamometer in response to an auditory “go” signal and offering a particular monetary incentive.	• Frontal, central, parietal • Task-related EEG	• Examined motor cortical beta suppression and motivation effects on effects on motor performance. • Beta-suppression was measured over motor cortical regions using 32-channel EEG during task-related motor preparation. • Motivation was manipulated with monetary incentives on each trial. • Both motivation and motor cortex beta-suppression independently influenced premotor reaction time. recorded during active task performance (motor preparation), not rest.
45	Mehrkanoon et al. (2016)	• TN = 20 • M = 10 • *F* = 10 • AR = 21.3 ± 1.8 years	• A dynamic force task required to make a transition 146 between two force levels as fast and accurately as possible	• Mu & beta bands • 112 anatomically defined regions of interest (ROIs) according to the macroscopic anatomical parcellation of the MNI template using the automated anatomic labeling (AAL) map • Resting state & Task-related EEG	• Motor skill acquisition involves critical interactions between the cerebellum and primary motor cortex. • Following a single session of motor training with a dynamic force task, resting-state functional connectivity was significantly upregulated mainly within the cerebellum and between the cerebellum and motor cortex. • These connectivity changes were specifically observed in the mu- and beta-frequency bands of brain activity. • An increased phase lag cerebellar activity after motor practice, indicating a reorganization of intrinsic cortico-cerebellar connectivity associated with motor adaptation. • The potential of EEG source connectivity analysis to reveal neural plasticity processes related to motor learning.
46	Mottaz et al. (2024)	• TN = 20 • M = 7 • *F* = 13 • AR = 28.7 ± 5.6 yrs	• Finger tapping task (FTT)	• Alpha (13–30 Hz) • Right primary motor & dorsolateral premotor cortex Right striatum (putamen and caudate nucleus, right Medial Temporal Lobe (MTL, including Hippocampus and Para hippocampus) • Resting state (pre & post-test), task-related EEG	• Sequence learning and consolidation were successful, shown by improved sequences per minute from pre-test to re-test. • Higher resting-state alpha-band functional connectivity (FC) in motor areas and medial temporal lobe (MTL) FC during training predicted better learning. • A decrease in beta-band FC in the MTL from before to after training also predicted learning. • Long-term expertise was linked to alpha-band FC in motor areas and striatum, beta-band FC during training, and years of piano playing. • Changes in striatum FC predicted consolidation. rather than local oscillations predicted learning, consolidation, and expertise, highlighting the importance of communication between brain regions.
47	Muthuraman et al. (2012)	• TN = 18 • M = 10 • *F* = 8 AR (20–52 yrs) = (mean: 29 ± 5)	• Finger movements, bilateral rhythmic hand movements, and isometric contractions	• Corticomuscular coherence in the 15–30 Hz band • Task-related EEG-EMG • Primary sensorimotor cortex (lateral areas). Premotor areas (particularly for higher-order coordination). Frontal midline region	• Significant corticomuscular coherence was found at the voluntary movement frequency or its first harmonic in all subjects • The 15–30-Hz coherence during isometric contractions was restricted to the contralateral cortex • Bilateral voluntary rhythmic movements were represented in a bilateral cortical network, including both sensorimotor cortices and occasionally premotor area • The strength of interactions between both hands' networks correlated with peripheral coupling
48	Nakano et al. (2013)	• TN = 20 • M = 16 • *F* = 4 • AR = 21.3 ± 2.3 yrs	• Ball rotation task in which two balls were rotated clockwise with the right hand	• Alpha −2 & Beta−2 bands • Parietal cortex (parietal lobe) & Frontal cortex • Task-related EEG	• The study results indicated that in addition to EEG activity during action execution, EEG activities during action observation and preparation were associated with motor skill improvement. • Specific decreases in alpha-2 and beta-2 EEG rhythms in parietal and frontal cortical areas during the observation, preparation, and execution of a motor task. This suggest the involvement of these brain rhythms in sensorimotor integration and motor control processes associated with action observation and execution. • Similar neural mechanisms are engaged during both watching and performing movements, highlighting the role of these EEG rhythms in motor learning and execution. • The functional significance of alpha and beta rhythm modulations in motor-related cortical areas for both observing and preparing/executing movements.
49	Nakayashiki et al. (2014)	• TN = 11 • AR = 19–23 yrs • Gender not defined	• Spatial correspondence between the stimulus & response locations • Close/open their right hand repetitively at three different speeds (Hold, 1/3 Hz, and 1 Hz) and four distinct motor loads (0, 2, 10, and 15 kgf). In each condition, participants repeated 20 experimental trials, each of which consisted of rest (8–10 s), preparation (1 s) and task (6 s) periods. Under the Hold condition, participants were instructed to keep clenching their hand (i.e., isometric contraction) during the task period.	• Mu & beta bands • Task- related EEG • Primary motor area C3/C4	• Repetitive hand grasping movements resulted in salient mu-ERD (8–13 Hz), & slightly weak slightly weak beta-ERD (14–30 Hz) in both hemispheres. • The strength of mu & beta-ERD was significantly weakened under the “Hold“ condition (isometric contraction), compared to the other speed and load conditions. • The strength of ERD may reflect the time differentiation of hand postures in motor planning or the variation of rather than the motor command itself.
50	Ozdenizci et al. (2016)	• TN = 21 • M = 14 • *F* = 7 • AR = 23.8 + 3.1 yr	• A force-field adaptation task involving 2D center-out reaching movements using a robotic handle.	• Beta band (15–30 Hz) the primary frequency band predictive of motor adaptation learning • Other bands studied (theta 4–7 Hz, alpha 8–14 Hz, gamma 55–85 Hz) but were not significantly predictive. • Resting-state EEG & Task-related • Sensorimotor cortical areas (two independent components, IC 5 and IC 6) • Fronto-parietal attention network (IC 1 and IC 4) • Parieto-occipital cortical region (IC 3, strongly predictive) • A subcortical source (IC 2) also contributed	• a distributed network of beta-frequency brain regions active both at rest and during task preparation preparation that predict and relate to motor adaptation learning in a force-field reaching task • Subjects with higher adaptation rates showed a significant decrease in pre-trial beta power in sensorimotor and while those with lower adaptation rates showed increases, indicating distinct EEG modulation patterns linked to modulation patterns linked to learning performance. • Resting-state beta power increased significantly after the motor learning task over the course of adaptation. • Motor learning relates to distributed cortical reorganization, not limited to primary motor areas.
51	Pangelinan et al. (2011)	• Total TN = 45 healthy right-handed females 3 groups (*n* = 15 each): • Young children mean ages 6–7 yrs • Older children 9–11 yrs • Adults 22.1 yrs	• A computerized, center-out visually guided aiming line drawing task • Participants made line-drawing movements from a central “home” position to one of two targets on the monitor with movement planned and executed as precisely and quickly as possible. Sixty discrete aiming trials were performed per participant	• Alpha (8–12 Hz) and Beta (13–30 Hz) bands • Frontal (F3, Fz, F4) • Central (C3, Cz, C4) • Parietal (P3, Pz, P4) • Occipital (O1, O2, though excluded from final analysis due to technical issues)	• Young children (6–7 y) showed: Less movement-related negativity (weaker MRCPs) in frontal/sensorimotor regions during planning and execution. • Greater task-related alpha desynchronization in frontal areas, less in parietal; reliance on frontal planning. • Slower, jerkier, and less consistent movement kinematics. • EEG and kinematics correlation: worse kinematics (slower/jerkier) were associated with higher frontal activation and lower sensorimotor area priming. • Older children and adults: • More efficient activation of sensorimotor areas (stronger MRCPs/C3 negativity). • Less frontal reliance, more distributed and parietal engagement. • Smoother, faster, and more consistent movements. • Functional EEG-behavior relationships show that age-related improvements in motor learning/kinematics are paralleled by more efficient, localized neural activation patterns
52	Penalver-Andres et al. (2022)	• TN = 36 • M = 22 • *F* = 14 • AR = 20–59 yrs	• Virtual surfing, requiring participants to steer a virtual boat using a joystick to surf waves as quickly as possible to a finish line following a resting-state EEG recording alternating between EO & EC conditions	• Alpha band • Resting-state EEG • Sensory-motor regions • Functional connectivity Somatosensory evoked potential (SEP) & vent-related desynchronization (ERD), • Effective connectivity by the phase slope index (PSI)	• Higher contribution of Microstate 3/C (posterior DMN) during resting-state was associated with poorer motor performance, indicated by longer completion times in the motor task • The presence of Microstate 4/D (AN) did not show a significant association with any behavioral metrics • Age & gaming/sailing experience were not significant confounding factors in the main findings
53	Perfetti et al. (2011)	• TN = 17 • M = 14 • *F* = 3 • AR = 21.2 ±1.4 yrs	• Participants were instructed to match the preprogrammed template while visual feedback was available	• Theta band (4–8 Hz); alpha band (8–12 Hz); low beta 13–18 Hz; high beta 18–30 Hz • Task-related EEG • Frontal midline regions (theta activity) • Central contralateral motor cortex areas (alpha and beta activity) • Frontal and electrodes show learning effects (theta changes) Central contralateral electrodes (alpha and beta desynchronization linked to success)	• Initial learning is associated with enhancement of gamma power in a right parietal region during movement execution as well as gamma/theta phase coherence during movement planning. • Stages of learning are instead accompanied by an increase of theta power over that same right parietal region during movement planning, which is correlated with the degree of learning and retention. • Gamma/theta phase coupling plays a pivotal role in the integration of a new representation into motor memories • Theta enhancement during planning and, adaptation and retention indices suggest that theta oscillations are involved in the sustained representation and retrieval of new internal models. • Theta band in reaching and motor learning and in memory acquisition, retention, and retrieval in verbal and spatial working memory tasks
54	Pitto et al. (2011)	• TN = 7 • M = 5 • *F* = 2 • AR = 24 ± 1 yrs	• 2D ball putting task • Participants grasped the handle of manipulandum & had to hit a virtual ball in order to put it into a target region (hole). • The robot was used to render the contact force with the ball during impact. At every trial, with respect to the initial ball position, the hole appeared in one of three different directions & two distances, selected randomly.	• Alpha, beta, theta bands • Resting state & task related EEG • EEG signals were recorded before and during each movement • Frontal midline regions (theta activity) • Central contralateral motor cortex areas (alpha and beta activity) • Frontal and fronto-central electrodes show learning effects (theta changes) • Central contralateral electrodes (alpha and beta desynchronization linked to success)	• EEG results showed increased frontal theta synchronization with practice. • Successful trials were preceded by higher theta synchronization and alpha and beta desynchronization. • EEG patterns suggest they can monitor motor learning progress and predict trial success or failure
55	Pollok et al. (2014)	• TN = 15 • M = 7 • *F* = 8 • AR = 28.0 ± 2.3 years	• Serial reaction time task • Oscillatory activity as a function of time was analyzed by calculating event-related desynchronization (ERD) individually for each condition	• Alpha (8–12 Hz) & beta band (13–30 Hz) • Primary sensorimotor cortex (S1/M1) • Resting-state MEG data Neuromagnetic activity was non-invasively recorded with a 306-channel whole-head MEG system • Since a pure resting baseline was not given, we defined the entire interval (−2, −3 s) as baseline.	• The correlation between beta suppression & improvement in reaction times • Subjects showing faster reaction times associated with stronger beta power suppression implicit learning might subjects using an explicit learning strategy rather prefrontal & premotor areas than M1 might be involved in motor learning and consolidation. • Involvement of the dorsal premotor cortex (dPMC) during early learning was shown
56	Quandt et al. (2016)	• Total TN = 66 • TN = 32 OA; M = 13; *F* = 19; AR = 61–81 yrs • TN = 34 YA; M = 20; *F* = 14; AR+ 19–34 yrs	• Finger sequence task (learning and executing a digit sequence). • Pinch grip and whole hand grip task (repetitive gripping).	• Alpha (8–13 Hz) and beta (13–25 Hz) bands • Sensorimotor cortex (contralateral and ipsilateral) • Premotor cortex • Supplementary motor areas • Frontal areas • Task-related EEG • EEG was recorded during the motor tasks (e.g., finger movement, pinch, hand grip). Baseline recordings were performed pre- and post-experiment at rest.	• Elderly participants showed more widespread and broadband power desynchronization during movement, with higher spectral entropy indicating less predictable • Young participants exhibited more distinct, peaked desynchronization in Alpha and upper Beta bands related to specific motor activities. • The increased spectral entropy in elderly suggests either less specialized neural coding or a compensatory, noise-like activity in their motor networks. • Elderly participants recruited a more extensive cortical motor network, involving bilateral primary motor areas, premotor, and supplementary motor areas. • The spectral distribution of motor oscillations differs with age, with broader, frequency-unspecific responses in older adults.
57	Ricci et al. (2019)	• TN = 26 • TN = 13 O A; M = 7; *F* = 6; AR = 57.5 ±8.2 yrs • TN = 13 YA; M = 3; *F* = 10; AR = 24.2 ± 4.5 yrs	• 30-min center-out reaching task (MOT): move a cursor with right hand to targets appearing randomly on screen using a digitizing tablet. Eight hundred and forty target presentations total (15 sets of 56 each)	• Beta band (15–30 Hz) • Left sensorimotor cortex (centered around C3 electrode) Frontal region (centered between Fz and F3 electrodes) • Task-related EEG EEG During the execution of the task, synchronized with behavioral events	• Investigates how Beta oscillatory activity (ERD/ERS and modulation depth) changes with task practice and reflects motor skill acquisition/plasticity in both younger and older adults. • Both younger and older adults showed a practice-related increase in beta modulation depth (larger difference between ERS and ERD) in left sensorimotor and frontal regions during the reaching task. • Older adults had slower and less accurate movements but exhibited similar beta modulation increases during practice as younger adults, suggesting plasticity-related beta changes are preserved with age. • ERS peak latency was delayed in older adults, correlating with total movement time, but overall magnitude of beta modulation did not differ. • No direct correlation was found between beta modulation magnitude and motor performance parameters, indicating beta oscillations reflect sensorimotor integration/plasticity rather than simple performance features.
58	Rogala et al. (2020)	• TN = 36 • M = 36 all male • 33 participants' data used for the visual search task • 23 participants' data for the shooting task after exclusions • AR = 21.97 ± 1.88 years	• Visual search task & shooting task	• Alpha & beta bands • Frontal, frontocentral, central, centroparietal, parietal, parietooccipital, & occipital regions • Resting-state & Task-related EEG	• The beta-2 band (22–29 Hz) activity was correlated with behavioral performance in both visual search & shooting tasks • Higher resting-state beta-2 power (gB2rest) is associated with longer reaction times & lower shooting scores • Weaker intrinsic frontoparietal & fronto-occipital connections are linked to better behavioral performance & greater capacity for network reconfiguration
59	Rosjat et al. (2024)	• TN = 27 • M = 16 • *F* = 11 • AR = 22–34 yrs	• Finger-tapping task	• Alpha band • Connectivity was analyzed across 150 parcels defined by the Destrieux atlas, focusing on regions relevant to motor and attention networks • Resting-state & Task-related EEG	• Four microstates were identified, showing no significant changes significant changes between pre-task (RS1) & post-task (RS2) resting states • Four connectivity states were identified, with CS, D serving as a transition state. The coverage of CS, C increased significantly
60	Rueda-Delgado et al. (2019)	• TN (YA) = 24 • M = 11 • *F* = 13 • AR = 21–32 yrs • TN (OA) = 24 • M = 14 • *F* = 10 • AR = 60–74 yrs (4 older adults excluded final gender distribution not defined)	• A bimanual coordination task	• Alpha & Beta bands • ROIs: were selected from the thresholded F-maps based on the local maxima (VMPFC: ventro-medial prefrontal cortex; PMV: premotor ventral; LTL: lateral temporal lobe; AMPFC: antero-medial prefrontal cortex; M1/S1: sensorimotor cortices; MTL: medial temporal lobe) • Resting state, task- related EEG	• Investigated how aging affects the neural mechanisms underlying the learning of a complex bimanual coordination task, as measured by changes in alpha & beta power in the brain. • Coordination level showed that age- related differences associated with learning occur across the spectrum of frequency bands. • Older adults showed lower task performance & less improvement in practice compared to younger adults. • Heterogeneous changes were observed in EEG spectral power with practice, with both increases and decreases observed in different brain regions & frequency bands, and these changes differed between the age groups. • Older adults started with higher levels of neural activity (lower spectral power) compared to younger adults, but ended up at similar levels after practice, likely due to greater decreases in spectral power in the younger group. This difference in neural activity patterns may explain the learning deficit in older adults.
61	Ryu et al. (2016)	• TN = 16 • M = 10 • *F* = 6 • AR = 24–31 yrs	• Cognitive task is a computer version of the Omok game • Motor task is a computer version of Alkkagi game (also a board game)	• Alpha, beta bands • Fz (frontal lobe), F3 (left frontal), F4 (right frontal lobe), Cz (central sulcus), C3 (center left), C4 (center right), P3 (left parietal lobe), and P4 (right parietal lobe) • Task-related EEG	• Greater frontal theta activity when subjects perform a cognitive task than in performing a motor task • No differences in alpha power and beta power between the two tasks.
62	Sallard et al. (2014)	• TN(YA) = 29 • M = 14 • *F* = 15 • AR = 19–29 yrs • TN (OA) = 27 • M = 12 • *F* = 15 • AR = 60–83 yrs	• Unimanual Tapping (UM) & Bimanual Tapping (BM)	• Low beta (14–20 Hz), & high beta (20–30 Hz) bands • Right premotor cortex (BA 6) • Right cingulate cortex (BA 24) • Left superior parietal lobule (BA 5) • Left occipital lobe • Task-related EEG	• Significant interaction between tapping condition & age group, with lower variability in BM than UM in young participants but not in the elderly • The elderly showed a decline in kinesthetic processing, leading to increased variability in tapping • Lower beta power was observed in the left superior parietal lobule during UM & in the left occipital lobe during BM in the elderly • Beta power was generally lower in BM than UM across both age groups
63	Schättin et al. (2016)	• TN = 30 but final = (27) • AR = 79.2 ± 7.3 yrs *Exergame group:* • TN = 13 • M = 8 • *F* = 5 • AR = 79.2 ± 7.3 yrs *Balance group:* • TN = 14 • M = 7 • *F* = 7 • AR = 79.2 ± 7.3 yrs	• Video game-based physical exercise: exergame tasks: included “Balloon,” “Step,” “Space,” & “Season“ games, which required participants to perform specific whole-body whole-body movements on a pressure-sensitive plate • Balance training tasks: included static & dynamic exercises on stable & unstable surfaces, performed in either bipedal or monopedal stance positions	• Total band width (1–30 Hz), delta (1–3.5 Hz), lower theta (3.5–5.5 Hz), upper theta (5.5–7.5 Hz), lower alpha (7.5–10 Hz), upper alpha (10–12.5 Hz), and beta (12.5–30 Hz). • Prefrontal cortex: the study focused on prefrontal brain activity, particularly in the Fp1 and Fp2 regions according to the 10–20 EEG system • Task-related EEG	• Theta relative power significantly decreased in the exergame group, which contrasts with the expected increase due to aging • The exergame group showed significant working memory, divided attention, go/no-go, and set-shifting, while the balance group only improved in set-shifting exergame group showed significant improvements in gait speed, cadence, & stride length during dual-task walking, while the balance group showed improvements primarily in single-task conditions
64	Studer et al. (2010)	• TN = 30 (after final exclusion 16 remains in total) • *F* = all female • AR = mean Age = 25.3 years, SD = 4.4 years	• continuous visuomotor tracking task	• Theta (4–8 Hz), Lower alpha (8–10 Hz), Upper alpha (10–12 Hz), Lower beta (12–20 Hz), and upper beta (20–30 Hz) • Task-related EEG with rest intervals • Sensorimotor cortex	• Frontal EEG activities in delta and theta bands of the whole trial Gamma band in the middle of the trial are having a significant negative relationship with the overall familiarity level of the task. • Performance Improvement: Both groups showed significant improvements in task performance over the course of practice • Task-Related Power Decrease: A reduction in Task-Related Power Decrease in the lower beta band was observed, indicating reduced motor-related cortical activation • Differences: The massed practice group showed a higher task-related theta power in later training blocks, indicating higher cognitive load & attentional demands compared to the distributed practice group
65	Sugata et al. (2020)	• TN = 53 • M = 31 • *F* = 22 • AR = 32.9 ± 6.7 yrs	• Sequential motor learning task (SRTT) & random button-press	• Alpha & beta bands • Primary motor area (M1), sensorimotor cortex: Superior temporal gyrus, opercular inferior frontal gyrus, parietal areas • Resting state & task-related EEG	• Negative correlations were found between beta-band rs-FC & motor learning index, particularly in the left superior temporal gyrus, bilateral sensorimotor areas, opercular frontal gyri & parietal areas • No significant correlations were observed between alpha-band rs-FC and motor learning index • Good & poor learners could be distinguished based on the strength of rs-FC between M1 & specific brain regions, with classification accuracy ranging from 70.1% to 76.0%
66	Titone et al. (2022)	• TN = 27 • M = 14 • *F* = 13 • AR = 23.6 +- 3.3 yrs Behavioral analyses: Task training *n* = 24 task retest *n* = 20 EEG analyses: pre-post *n* = 21 Correlation with online gains *n* = 18 Correlations with offline gains *n* = 15	• Digital version of the classic pursuit motor task motor learning paradigm • An explicit bimanual finger-tapping task implemented in MATLAB with Psychophysics Toolbox version 3	• Alpha, beta, gamma & bands • The 21 chosen ROI were defined in the Montreal National Institute (MNI) space & then projected to individual space. • Parietal regions • Resting-state EEG	• The strength of the gamma-band connectivity at rest would also be related to motor performance. • Brain-behavior correlation analyses revealed that baseline beta, delta, and theta rsFC were related to subsequent motor learning and memory consolidation such that lower connectivity within the motor network and between the motor and several distinct resting-state networks was correlated with better learning and overnight consolidation. • Training-related increases in beta-band connectivity between the motor and the visual networks were related to greater consolidation.
67	Tzvi et al. (2016)	• TN = 73 • M = 36 • *F* = 37 • AR = 18–31 yrs	• Serial reaction time task (SRTT)	• Theta (4–8 Hz), Alpha (8–13 Hz), and low Gamma (30–48 Hz) • Central electrodes, sensorimotor event-related desynchronization (ERD) • Resting-state & Task-related EEG	• Larger alpha power was observed over posterior parietal areas during the first learning session (SES1), which decreased in later sessions (SES3) • Theta power was during early learning over parietal areas and decreased in later sessions • Increased gamma power was found over right parietal areas during initial learning stages • Reduced alpha/low-gamma PAC was observed over right parietal and bilateral frontal cortex during learning
68	Usanos et al. (2020)	• TN = 12 • M = 3 • *F* = 9 • AR = 21–47 yrs	• Pursuit tracking task basal recording: a total of 18 min of basal activity are recorded, divided into 3 parts (each 6 min long) with a rest of approximately 1 min between each. • Imaginary motor task: the imaginary task consists of simulating, without muscle activation, rapid extension of the wrist followed by brief relaxation. This phase lasts approximately 40 min • Actual motor task: the same characteristics and duration of imaginary motor task.	• Gamma band activity (GBA) • Cerebral motor areas • Task-related EEG	• ERS could provide a useful way of indirectly checking the function of neuronal motor circuits activated by voluntary movement, both imaginary and actual. These results, as a proof of concept, could be applied to physiology studies, brain–computer interfaces, and diagnosis of cognitive or motor pathologies.
69	Van Der Cruijsen et al. (2021)	• TN = 20 • M = 7 • *F* = 13 • AR = 18–30 yrs	• Sequential visual isometric pinch tasks in a counterbalanced order: a complex motor task (CMT) that has been shown to induce learning over many repetitions and a simple motor task (SMT) which required little to no learning over repetitions.	• Theta (5–8 Hz) and alpha band (8–12 Hz) • Task-related EEG • M1, CC regions, aMCC, anterior mid-cingulate cortex; cM1, contralateral primary motor cortex; iM1, ipsilateral primary motor cortex; pMCC, posterior mid-cingulate cortex; PCC: posterior cingulate cortex.	• Contralateral primary motor cortex (cM1) theta & alpha power, but not beta power, are positively associated with motor learning • Theta & alpha power in the posterior mid-cingulate cortex (pMCC) & posterior cingulate cortex (PCC) are also positively associated with motor learning. • No association between M1 beta power and learning, but the CMT produced stronger bilateral beta suppression compared to the SMT. • Positive association between contralateral M1 theta (5–8 Hz) & alpha (8–12 Hz) power & motor learning, & theta & alpha power in the posterior mid-CC and posterior CC were positively associated with greater motor learning. • M1 beta power is associated with the capability of performing a motor task, but not with learning the task.
70	Veldman et al. (2017)	• TN = 24 • M = 10 • *F* = 14 • AR = 21.0 ± 1.7	• visuomotor task • After randomization, participants performed a motor practice (MP) intervention in a visuomotor skill for 15 min with the right-dominant hand (*n* = 12) or rested for 15 min (Control, *n* = 12). • On Day 1 of two visits on consecutive days, participants performed baseline measurements while EEG was recorded including resting-state EEG, ERD, and N30 SEPs, became familiarized, & were tested for the visuomotor task with each hand. • Immediately (Day 1) and 24 h (Day 2) after MP or Control, we repeated baseline measurements to determine the immediate & delayed effects of visuomotor practice on motor performance and EEG measures.	• Alpha & beta bands • Resting state & task-related EEG	• The algorithm practice, due to optimal activity in the frontal lobe (medium alpha & beta activation at prefrontal), resulted in increased activity of sensorimotor areas (high alpha activation at C3 and P4) in older adults. • Similar conditions could affect the intertrial interval period (medium alpha and beta activation in frontal) while the dissimilar conditions could affect the motor period (medium alpha and beta activation)
71	Veldman et al. (2021)	• TN (YA) = 24 • M = 10 • *F* = 14 • AR = 18–24 yrs • TN (OA) = 24 • M = 11 • *F* = 13 • AR = 65–87 yrs	• The visuomotor task consisted of following a template using right- & left wrist flexion & extension movements	• Alpha & beta band • Resting state & task- related EEG • Sensorimotor regions, left M1 & right M1	• Both younger and older adults showed similar right-hand skill acquisition and consolidation, no significant age differences for the trained hand interlimb transfer to the left (untrained) hand was reduced in older adults compared to younger adults. • EEG connectivity focused on the beta band (13–30 Hz) involving the primary motor cortex. • Older adults showed age-dependent modulation in bilateral motor network connectivity
72	Vieluf et al. (2018)	• TN of young novices = 14 • M = 5 • *F* = 9 • AR = 20–26 yrs • TN of late middle-aged novices = 12 M = 5 • *F* = 7 • AR = 57–67 yrs • TN of late middle-aged experts = 14 • M = 6 • *F* = 8 • AR = 57–67 yrs	• Force maintenance task: Precision grip task	• Alpha band (8–13 Hz) and also low beta (13–20 Hz), high beta (20–30 Hz), and theta (4–8 Hz) bands • Resting-state & task-related EEG • Sensorimotor regions, the frontal & occipital regions	• Late middle-aged novices showed more variable and less complex force control performance than young novices and experts. • A decrease in overall network activity was observed in the alpha band (8–13 Hz). • Aging leads to deterioration in force control reflected in behavioral variability and neural network activity. • Deliberate practice can age-related declines by modifying sensorimotor and attentional brain networks.
73	Wang et al. (2022)	• TN = 21 • M = 11 • *F* = 10 • AR = aged 23.29 ± 3.47 yrs remained and defined: 15 participants (8 females; age 22.73 ± 2.69 years old	• Functional motor task focused on reaching & fine motor control • Participants use their non-dominant hand to transfer beans from a central “home” cup to three target cups, completing 15 reaches completing 15 reaches per trial. • Performance is measured by elapsed time, with only five training	• Alpha & Beta bands • Contra- and ipsilateral frontal cortex, motor cortex & parietal cortex • Resting state 2-min eyes closed, then Visuospatial/Constructional Index (RBANS) the Assessment of Neuropsychological Status (RBANS) then motor task in 5 trials	• Age-related deterioration in motor performance was more pronounced with increasing task difficulty & was accompanied by a more bilateral activity pattern for older vs. younger adults. • Task difficulty affected motor skill retention & neural plasticity specifically in older adults. • Older adults that practiced at the low or medium, but not the high, difficulty levels were able to maintain improvements in accuracy at retention & showed modulation of alpha after practice.
74	Wong et al. (2013)	• TN = 38 • M = 21 • F = 17 • AR = 22.1 ± 2 yrs	• Performed 8 trials of a computerized visual–motor task similar to mirror drawing • Trace within the boundary of the tetradecagon (within the boundary) with a with a computer mouse. • Participants were given no longer than 301 s to complete the tracing.	• Delta, theta, alpha, gamma & beta bands • Theta (4–8 Hz), Lower Alpha (8–10 Hz), Upper alpha (10–12 Hz), Lower Beta (12–20 Hz), and Upper Beta (20–30 Hz) • Task-related EEG • Prefrontal cortex & primary motor cortex	• EEG power spectra decreased with an increase in motor task familiarity. • Frontal EEG activities in delta & theta bands of the whole trial & in gamma band in the middle of the trial are having a significant negative relationship with the overall familiarity level of • Frontal EEG spectra are significantly modulated during motor skill acquisition. • Possibility of simultaneous monitoring of brain activity during an unconstrained natural task with a single dry sensor mobile EEG in an everyday environment
75	Wu et al. (2014)	• TN = 17 • M = 9 • *F* = 8 • AR = 22.1 ± 3.0 years (18–30 yrs)	• digital version of pursuit rotor task	• Beta band • Resting-state & task-related EEG • Primary motor cortex, parietal cortex, & frontal-premotor areas	• Practice was associated with task performance • Partial least squares regression (PLS) model, coherence with the region of the left primary motor area (M1) in resting EEG data associated to a strong predictor of motor skill acquisition • EEG coherence can predict individual motor skill acquisition with a level of accuracy that is remarkably high
76	Wu et al. (2017)	• TN = 32 • M = 14 • *F* = 18 AR = 19.4 ± 1.6 yrs, 18–30 yrs	• Serial reaction time task • Performed the task using the dominant, right hand. • Wrist flexion resulted in the cursor moving left on the screen & wrist extension resulted in the cursor moving right on the screen. • To maximize consistency of movements across two soft straps were to the participants' right forearm to limit elbow movement. • To standardize task difficulty, cursor movements were normalized to each participant's active range of motion at the wrist.	• High Beta (20–30 Hz) • left M1 & left dorsal prefrontal cortex (dPF), left lateral parietal cortex (latPAR) & left sensory cortex (S1). • Resting-state & task-related EEG	• Individual differences in brain connectivity, particularly in the premotor and motor cortices, significantly correlate with training-induced improvements in performance on the motor sequencing task. • Higher coherence in specific frequency bands, especially in the beta range, is associated with better learning outcomes.
77	Yang et al. (2017)	• TN = 1 • M = One healthy adult male • AR = aged 20	• Continuous tracking task • One participant performed 16 trials of the continuous tracking task with different constant patterns on Day-1 & Day2 with a three-day interval.	• Alpha band: Individual Alpha Band (IAB), subdivided into Low IAB (LIAB) and High IAB (HIAB), analyzed due to individual variation. Beta1 (16–20 Hz) Beta2 (20–28 Hz). Other analyzed bands: Delta (0.5–4 Hz), Theta (4–8 Hz), Sigma (12–16 Hz). • Resting- state & task-related EEG • Left primary motor cortex (C3 electrode) bilateral parietal areas (P3, P4) and frontal areas (F3, F4). occipital (O1, Oz, O2), central (Cz, C4) • The EEG signals from 16 channels in both the resting and active states were recorded, & the relative EEG amplitudes & the EEG coherence between the left primary motor cortex & other brain regions in eight frequency frequency bands were analyzed	• Motor tracking performance improved significantly from Day 1 to Day 2, indicating motor learning and consolidation. EEG relative amplitudes in several frequency bands differed resting and active states. High-frequency bands (sigma, beta1, beta2; 12–28 Hz) showed increased amplitude in the active motor state, linked to motor cortex excitability. Alpha band (individual alpha band, IAB, and its sub-bands LIAB and HIAB) amplitudes decreased locally over the left primary motor cortex during the active state but increased in other brain areas.
78	Yordanova et al. (2020)	• TN = 21 • TN (YA) = 10 • *F* = 5 • M = 5 • AR = mean age 22.5 ±1.5 years • TN (OA) = 11 • *F* = 6 • M = 5 • AR = mean age 58.3 ± 2.1 years)	• Four-choice reaction task (CRT) (auditory and visual)	• Theta band (3.5–7 Hz) • Task-related EEG • Frontal cortex & sensorimotor cortex regions of interest including bi-lateral electrodes were used –fronto-central, central & centro-parietal (FC5/6, FC3/4, C5/6, C3/4, CP5/6, CP3/4). • Midline electrodes (Fz, FCz, Cz, CPz and Pz) were included in separate analyses	• Aging was associated with a significant reduction in midline frontal-central theta power & a reduced functional asymmetry in theta synchronization for left-hand responses • Theta oscillations were phase-locked to the response onset at motor cortical regions contra-lateral to the responding hand in both age groups • Older adults showed a suppression of medial frontal theta power during correct response generation
79	Yordanova et al. (2024)	• TN = 27 • TN (YA) = 14 • *F* = 5 • M = 9 • AR = mean age 22.5 years • TN (OA) = 13 • *F* = 6 • *M* = 7 • AR = 59–70 mean age 58.3 years	• Simple Reaction Task (SRT) • Go-NoGo task Four-Choice Reaction Task (CRT) in two modalities, auditory & visual	• Theta band • Task-related EEG • Response-related theta oscillations were computed. • The phase-locking value (PLV) was used to analyze the spatial synchronization of primary motor & motor motor control theta networks.	• In older adults, synchronization was limited to intra-hemispheric regions only, indicating a functional decoupling between the motor cortex and higher motor control regions in the regions in the theta band. • Specifically, in younger adults, the contralateral primary motor cortex was synchronized with motor control regions (intra-hemispheric premotor/frontal and medial frontal areas). • Reaction times were slower in older adults, especially in the CRT task
80	Zhang and Fong (2019)	• TN = 18 • *F* = 9 • M = 9 (Final TN is 16) (Final gender distribution not defined) (Group 1 = 5, Group 2 = 6, Group 3 = 5) • AR= 18–30	• Effects of intermittent theta burst stimulation (iTBS) with mirror training (MT) on mirror visual feedback (MVF)-induced sensorimotor brain activity and motor performance assessed with • Nine-hole peg test (NHPT) • The Minnesota dexterity test (MDT) • The Purdue Pegboard Test (PPT) • The two-ball rotation task	• Mu & beta band • Mu-1 (8–10 Hz) • Mu-2 (10–12 Hz) • Beta-1 (12–16 Hz) • Task-related EEG (Group 1: iTBS + MT, Group 2: iTBS + sham MT, Group 3: sham iTBS + MT).	• Combined effects of Intermittent theta burst stimulation (iTBS) & mirror training (MT) enhanced the brain's responsiveness in MVF and MVF induced a shift of sensorimotor ERD toward the contralateral hemisphere in mu-1, mu-2, and beta-1 bands • Visual feedback MVF is likely to activate the contralateral sensorimotor cortex, however it can't translate into significant improvements in motor performance.

Across the reviewed literature, neural oscillations across several frequency bands, including alpha, beta, theta, mu, and gamma were found to correlate with improvements in motor learning. Many studies reported significant associations between EEG activity and behavioral performance during motor training, which are examined in more detail within the respective frequency-specific subsections of this review.

To better synthesize findings across diverse studies, we categorized all included studies by dominant EEG frequency band, even if the study's primary focus was on other factors such as age or recording condition (e.g., resting state vs. task-based EEG). This classification approach helps clarify how each band contributes to motor learning across various experimental conditions. A key theme emerging from several studies is the role of age as a potential modulator of motor learning success, with implications for e.g., peak performance, neurorehabilitation and AI assistive technologies. Our review highlights findings that explore whether age should be considered a crucial variable when developing interventions or when evaluating the robustness of motor learning across different life stages throughout basic or clinical research. For example, Christov and Dushanova (2016) investigated how aging affects brain performance by examining beta and gamma components of event-related potentials (ERPs) during an auditory discrimination task. They concluded that aging disproportionately impacts cognitive functions compared to sensory processing, with notable reductions in higher-frequency brain activity.

Deiber et al. (2014) examined mu and beta band activities during motor preparation and execution. Their findings emphasize age-related functional reorganization, with older adults displaying a shift in responsiveness from mu to beta frequencies. This supports the dedifferentiation hypothesis, which suggests that aging leads to less efficient use of neural resources and compensatory recruitment of additional circuits. Their results revealed increased beta-range activity and greater cortical activation during motor tasks, indicative of compensatory or reorganizational processes in older adults.

Further evidence from Yordanova et al. (2020, 2024) revealed that theta oscillations were phase-locked to motor response onset in both young and older adults. However, aging was associated with reduced midline frontal-central theta power, diminished functional asymmetry in theta synchronization, and slower reaction times, particularly during complex motor tasks (e.g., choice reaction tasks). These findings suggested a reorganization of motor theta networks in aging, characterized by increased intra-hemispheric and decreased inter-hemispheric connectivity.

In support of the alpha inhibition theory, Bönstrup et al. (2015a) found that elderly participants (aged 65+) exhibited no significant post-learning increase in alpha power in the primary motor cortices, unlike their younger counterparts. This points to a reduced capacity for inhibitory control in older adults, likely due to altered connectivity between frontal executive and sensorimotor networks. Notably, alpha-related inhibitory rhythms were enhanced following overnight consolidation in young adults but remained attenuated in older participants, suggesting diminished motor memory trace consolidation in aging.

Structural and functional brain connectivity also plays a critical role. For instance, Babaeeghazvini et al. (2018) examined how age-related differences in motor network connectivity (via resting-state and task-related EEG) relate to bimanual motor performance. Their study found that weaker structural connectivity between the dorsal premotor cortex (PMd) and primary motor cortex (M1) in the left hemisphere was associated with stronger—but less efficient—functional connectivity, ultimately correlating with poorer motor performance in older adults. These findings highlighted the importance of both anatomical and functional network integrity for effective motor learning across the lifespan.

#### 3.4.2 Age, expertise, and neural dynamics in motor learning

Age-related changes in motor control have been extensively documented in the literature. Vieluf et al. (2018) specifically highlighted that aging is associated with a decline in the efficiency of neural control mechanisms, which often manifests as reduced force control capabilities. These findings are consistent with prior studies (Spedden et al., 2019; Roski et al., 2013), which showed age-related reorganization of sensorimotor networks. In particular, older adults frequently demonstrated greater cortical activation during simple motor tasks, suggesting compensatory recruitment of additional neural resources to offset declining motor efficiency.

Moreover, older adults tend to rely more heavily on cognitive resources during motor execution, leading to increased variability and greater task difficulty (Vieluf et al., 2018). Frolov et al. (2020) reported that elderly participants show significant delays in motor initiation, attributed to altered cortical activity patterns and increased sensorimotor coupling during the pre-movement phase. These findings underscore a shift from efficient motor planning to compensatory sensorimotor integration strategies with age.

#### 3.4.3 The role of expertise in sensorimotor efficiency

Beyond age, motor expertise also plays a critical role in shaping neural control mechanisms. Vieluf et al. (2018) reported that individuals with expertise in fine motor tasks (e.g., precision mechanics) display enhanced force control and sensorimotor modulation compared to novices. These enhancements are likely underpinned by greater connectivity and coordination among motor-related brain regions, as observed in individuals with extensive training and task-specific experience.

Utilizing a dynamical systems approach, Morrison and Newell (2012) analyzed the variability in force control, identifying both deterministic and stochastic components contributing to performance differences across age and expertise. These insights emphasize the necessity of considering both age and experience when evaluating neural strategies in motor tasks.

#### 3.4.4 Neural correlates of motor learning: EEG frequency bands

Studies utilizing EEG have consistently highlighted the importance of brain oscillations in motor learning, particularly in the beta frequency band (13–30 Hz). Beta activity is implicated in multiple facets of motor function, including preparation, execution, and post-movement states. For instance, event-related desynchronization (ERD) in the beta band precedes movement and reflects motor readiness, while post-movement beta rebound (PMBR) indicates the brain's return to a resting state (Abbasi and Gross, 2019; Haar and Faisal, 2020a,b). Research also links beta oscillations with feedback integration and error correction, especially in tasks requiring visual feedback (Barone and Rossiter, 2021; Davis et al., 2012). Meadows et al. (2016) demonstrated that beta suppression in the contralateral motor cortex correlates with faster reaction times, reinforcing its role in preparatory neural states.

Beta-band coherence has further been associated with functional connectivity and motor inhibition control. Wu et al. (2014) identified beta oscillations (20–30 Hz) as strong predictors of motor skill acquisition, outperforming other bands such as theta, alpha, and gamma. Moreover, Ding et al. (2023) suggested that elevated beta power correlates with diminished motor inhibitory control.

In the context of resting-state functional connectivity, Sugata et al. (2020) found significant correlations between beta-band rs-FC and motor learning capabilities, particularly involving the M1 seed region and other motor-relevant brain areas. By contrast, alpha-band rs-FC did not show such associations.

#### 3.4.5 Beyond beta: other relevant frequency bands

Although beta rhythms dominate the motor learning literature, other frequency bands also contribute to motor learning:

- Delta Band (0.5–4 Hz), though less studied, delta oscillations are implicated in motor planning and directional control, possibly modulating higher-frequency activity (Hamel-Thibault et al., 2016).
- Theta Band (4–8 Hz) is particularly relevant for implicit sequence learning and motor-cognitive integration (Schättin et al., 2016; Van Der Cruijsen et al., 2021; Yordanova et al., 2020, 2024). Theta activity at midline-frontal and parietal sites has been linked to enhanced learning and cortical plasticity.
- Low Frequency Bands (2–5 Hz), Anwar et al. (2016) identified this band using EEG-EMG coherence, linking it to activity in the PPC, MFC, and PFC during finger movement tasks. Moreover, prior research (Muthuraman et al., 2012) has shown that corticomuscular coherence between bilateral cortical areas is crucial for coordinating bimanual movements. Different motor rhythms, particularly in the beta frequency band, exhibit interhemispheric coherence and generate synchronized motor activity that enables effective communication between the two hemispheres. This synchronization supports both the execution and learning of bimanual movements by facilitating interhemispheric communication necessary for smooth and coordinated actions. Importantly, such coherence is dynamically modulated during task execution, reflecting the changing demands of coordination between the two hands. Alpha Band (8–12 Hz), associated with attention and sensorimotor integration, alpha activity has been shown to predict motor-skill acquisition (Allaman et al., 2020; Rosjat et al., 2024). Alpha modulation is often observed during both task-related and resting-state conditions and is crucial for understanding changes in connectivity during motor learning (Bootsma et al., 2020; Mottaz et al., 2024).
- Mu Band (11–14 Hz) is closely related to beta, mu rhythms are prominent in sensorimotor regions and show ERD/ERS patterns during motor tasks (Nakayashiki et al., 2014; Deiber et al., 2014). These bands are essential in motor preparation and feedback processing.
- Gamma Band (30–100 Hz): Despite limited findings, gamma rhythms may play a role in fine motor control and coordination (Amo et al., 2015, 2017; Hamada et al., 2023; Usanos et al., 2020).

#### 3.4.6 Oscillatory modulation through external training

Recent studies explored methods to enhance motor learning through external modulation:

- Visuo-Tactile Feedback: Jahanian Najafabadi et al. (2023) found that multisensory integration improves motor learning, with differential patterns of alpha, beta, and theta activity in older vs. younger adults.
- Binaural Acoustic Beats (BAB): Herozi et al. (2024) demonstrated that alpha BAB enhances motor performance by increasing oscillatory activity across different bands in young and older adults, albeit with distinct neural signatures.

From these studies, we learned that the extensive body of research on brain oscillations reveals their critical relevance as biomarkers for motor learning prediction, highlighting how various EEG frequency bands especially beta, alpha, theta, mu, and gamma reflect the neural dynamics underlying motor skill acquisition and control. Beta oscillation (13–30 Hz), has received most of its attention in the motor learning literature, being strongly linked to motor readiness, execution, feedback integration, and post-movement states, with changes in beta power correlating with motor performance and plasticity. Age has a substantial impact on motor learning-related neural circuits; older individuals typically show less flexible reorganization (in alpha and beta bands), greater levels of beta power during rest, changing connectivity in the motor network, and reliance on compensatory recruitment of neural circuits, resulting in poorer motor efficiency when learning a new task, and generally slower learning. Theta oscillations play a role in motor-cognitive integration and implicit learning, while alpha rhythms are crucial for attention and sensorimotor integration, with reductions in alpha power post-learning and during consolidation noted in older adults, with moderate gains also reported diminishing by experience. Other neuromodulation approaches such as visuo-tactile feedback and alpha binaural acoustic beats have shown promise in enhancing motor learning by modulating these oscillatory activities, additionally such interventions can yield different results across age groups. This integrated evidence supports the utility of brain oscillations as predictive markers of motor learning capability and provides the necessary groundwork to support the development of age-sensitive interventions for neurorehabilitation and the use of neuro-enhancement strategies to support optimal motor skill acquisition throughout our lifespan.

#### 3.4.7 Conflicting and converging findings across studies

The literature on oscillatory brain activity and motor learning has been inconsistent with respect to its ability to enhance motor learning through oscillatory brain activity. For example, one set of studies noted that changes in local oscillatory power, specifically in the theta and high-gamma phase-amplitude coupling, were essential during motor skill acquisition, emphasizing task-specific modulation within motor cortical areas. Others argued that local oscillatory power changes are not adequate as predictors for subsequent motor learning. Instead, those studies emphasized the dynamic interconnectedness of large-scale functional connectivity dynamics across alpha and beta bands spanning multiple brain regions, including the motor cortex, striatum, and medial temporal lobe (Mottaz et al., 2024). Furthermore, there is variability across studies on the oscillatory frequency bands that are included. For example, beta oscillations can be specific to the maintenance of motor states and thus are always associated with motor learning (e.g., Dyck and Klaes, 2024; Khanjari et al., 2023; Sallard et al., 2014; Matta et al., 2025). Whereas, other studies suggested that the motor learning process itself included either alpha or theta bands only (Studer et al., 2010; Van Der Cruijsen et al., 2021). The variances from prior studies might be due to differences in the experimental paradigms, neural recording modalities, and participant populations.

Although inconsistencies persist, there is some agreement when the studies were put into the context of certain contextual factors such as motor task type, or their research purpose. Research that employs simple tasks like finger-tapping, or serial response paradigms generally emphasize the role of cross-frequency coupling (e.g., theta–high gamma) specifically focused within the motor-related cortical areas during the preliminary stages of learning (Dürschmid et al., 2014). More complex forms of motor sequence learning tasks typically engage interactions not only in local oscillatory activity, but also network-level interactions, specifically functional connectivity in the alpha and beta frequency bands. In addition, the researchers' purpose of research was to assess immediate motor performance, long-term consolidation, or discovery of underlying neural mechanisms shapes which oscillatory features are most notable. In some studies, authors emphasized on using clinical populations, while applying clinical interventions (inducing frequency band modulation) like transcranial alternating current stimulation (tACS) or fMRI to present changes in performance or symptoms. The results of these studies may also vary due to clinical heterogeneity (Takeuchi and Izumi, 2021).

#### 3.4.8 EEG studies by age group and frequency bands

**Figure 3:**
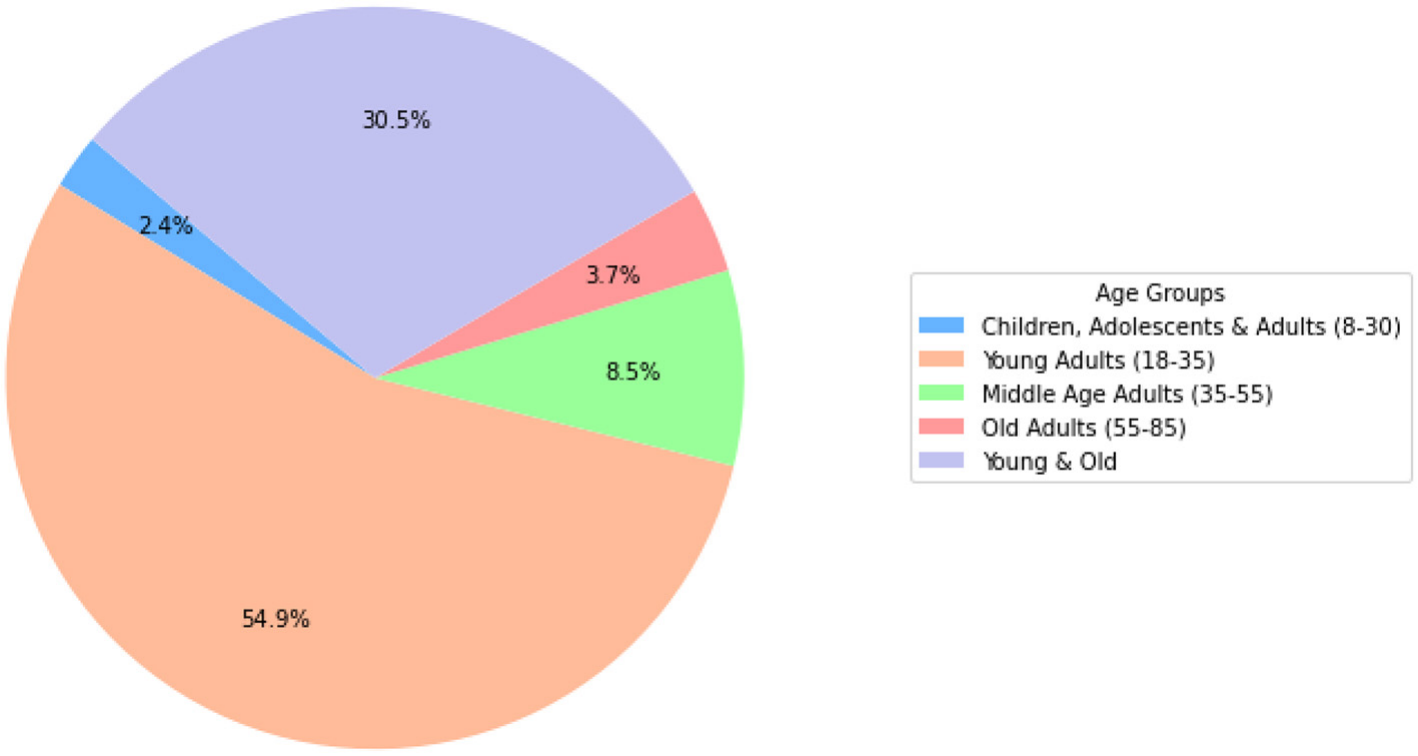
Age group distribution in motor learning studies.

**Figure 4:**
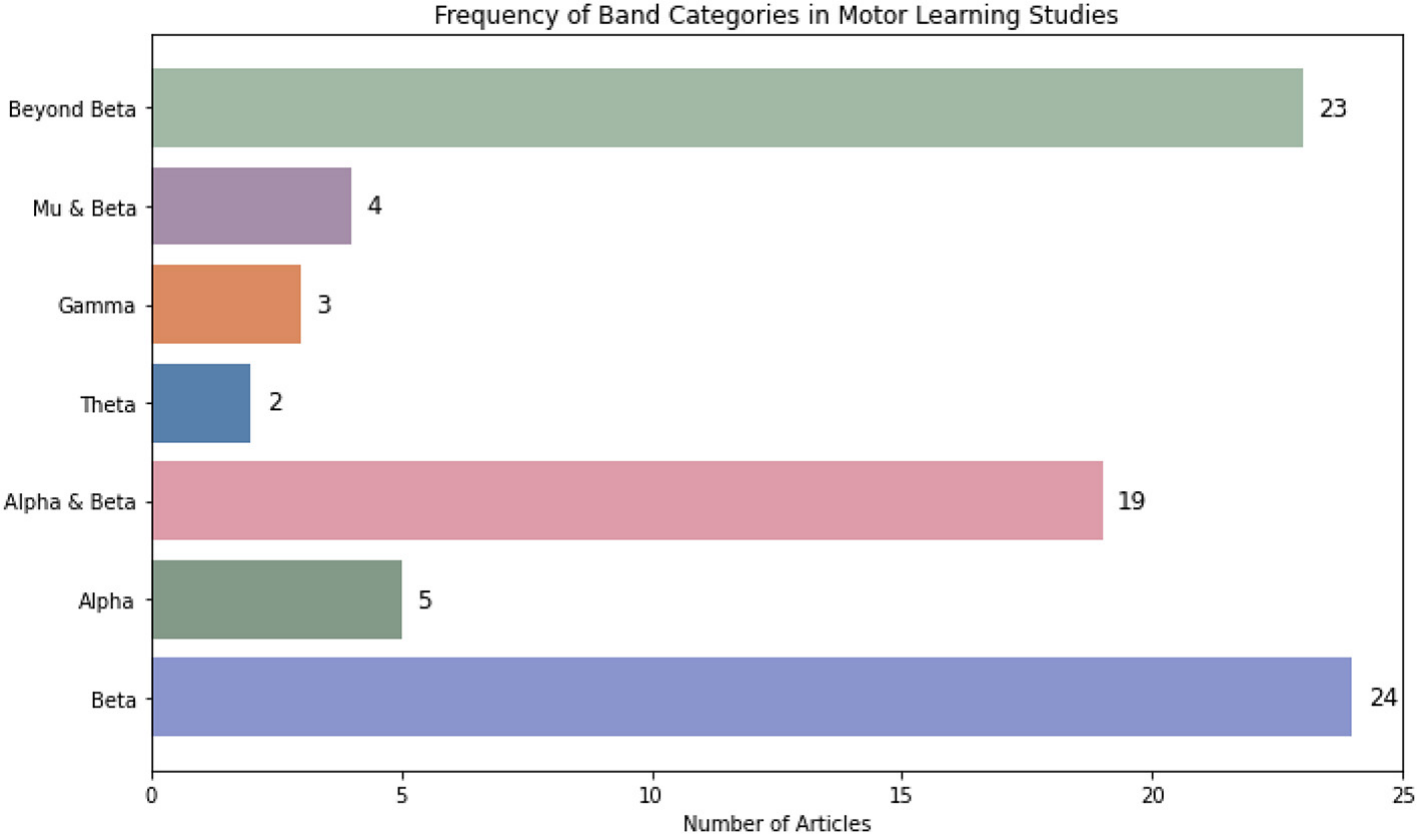
Frequency band distribution in motor learning studies.

In Table 1, we present a summary of studies categorized by the frequency bands explored. Of the 80 studies reviewed, 45 focused on participants in the young age group (18–35 years), 7 of those are in the middle-aged group (35–55 years), and 3 from the older age group (55–85 years). Additionally, 25 studies included participants from both the young and older age groups. See Figures 3, 4 for graphical information and Table 1 for detailed frequency bands per age group reported by reviewed studies.

#### 3.4.9 Age-related findings across studies

Table 1 summarizes studies that compared age-related data across three groups: Young Adults (YA; 18–35 years), Middle-aged Adults (MA; 35–55 years), and Older Adults (OA; 55–85 years). Abbreviations: YA = Young Adults; MA = Middle-aged Adults; OA = Older Adults.

#### 3.4.10 Reviewed studies across adulthood life span

In the following table, we present participants' demographic, task type, frequency bands and other related parameters and results reported by each study. Therefore, participants age and gender demographics indicated as: TN = total number of participants, F = female, M = male, AR = age range, yrs = years, YA =young adults, OA = old adults.

## 4 Discussion

In this systematic review, we synthesized findings from 80 experimental studies examining the relationship between motor learning and brain oscillatory activity, with a specific focus on age-related differences across the adult lifespan. Our objective was to clarify how resting-state and task-based neural oscillations relate to motor skill acquisition, retention, and adaptation, and whether these patterns vary meaningfully with age. Building on the hypothesis that aging modulates the neural mechanisms underlying motor learning, we aimed to identify oscillatory markers that may predict individual learning outcomes and offer insights into compensatory processes in older adults. Overall, the evidence reviewed highlights several consistent patterns. First, motor learning is preserved in older adults, though modulated by age-related changes in neural efficiency and connectivity. Second, beta and alpha oscillations consistently emerge as critical predictors of motor learning across age groups (Dyck and Klaes, 2024; Mottaz et al., 2024). Third, the engagement of broader cortical networks in older adults suggests compensatory recruitment rather than fundamental deficits. These findings suggest that despite reduced processing efficiency, the aging brain retains a remarkable capacity for adaptation (Van Ruitenbeek et al., 2022).

A growing body of research has investigated how aging alters cortical dynamics during motor learning. Declines in motor performance with age are often attributed to less efficient neural communication and changes in functional connectivity within motor control networks. Notably, older adults tend to experience greater performance drops as task demands increase (Seidler et al., 2009; King et al., 2013, 2017; Bootsma et al., 2021; Herozi et al., 2024; Rueda-Delgado et al., 2019). However, despite these challenges, the ability to acquire new motor skills is largely preserved in later life. This suggests that age-related plasticity remains active and may be supported by alternative or compensatory neural strategies. Factors such as task complexity, feedback type, and learning conditions appear to influence how motor learning unfolds in older adults (Veldman et al., 2021; Sallard et al., 2014), underscoring the need to account for these variables when interpreting age-related differences in motor learning performance.

Brain oscillations measured through EEG are often associated with learning and neuroplasticity. However, it is important to recognize that these oscillations are correlated —not directly measures— plastic changes. While oscillatory activity may reflect processes involved in learning and adaptation, its precise role remains an area of active investigation. Beta oscillations (13–30 Hz) are especially important in motor learning. Typically seen in sensorimotor and frontal areas during wakefulness, beta rhythms are thought to support top-down processing, sensory integration, and the maintenance of current brain states (Engel and Fries, 2010; Spitzer and Haegens, 2017). They play a key role in motor preparation and execution, as well as in integrating sensory feedback. Multiple studies have identified beta oscillations as central to motor learning, especially in the frontal, parietal, and temporal lobes, as well as the basal ganglia and sensorimotor cortex (Mottaz et al., 2024; Hamada et al., 2023; Espenhahn et al., 2019; Barone and Rossiter, 2021).

The beta rebound—an increase in beta power following movement completion—serves as a marker for movement termination (Studer et al., 2010). During motor learning, baseline beta activity tends to decrease, while modulation in response to motor tasks becomes more dynamic (Boonstra et al., 2007; Houweling et al., 2009). Baseline beta power has even been proposed as a predictor of subsequent learning and consolidation processes (Titone et al., 2022). Some studies have also observed reduced beta coherence after training (Ghasemian et al., 2017). While several investigations have noted increased beta power at rest in older adults (Heinrichs-Graham et al., 2018), others found no significant age-related differences (Babiloni et al., 2006). These patterns may reflect age-related compensatory mechanisms which have been attributed to the change in alpha and beta patterns in aged participants, wherein this age groups engage additional motor and prefrontal resources to maintain performance, aligning with the Scaffolding Theory of Aging and Cognition (STAC, Park and Reuter-Lorenz, 2009; Knights et al., 2021; Derya and Wallraven, 2024). Advancing age has been associated with changes in the default mode network (Duda et al., 2019), characterized by increased activation in frontal brain regions and reduced activation in posterior areas. This shift is commonly interpreted as a compensatory mechanism to counteract age-related decline in specific neural systems (Reuter-Lorenz and Park, 2010). Such neural alterations significantly influence functional reorganization in older adults compared to younger individuals, resulting in modified patterns of cognitive, sensory, and motor processing. These changes can impact performance across a range of everyday activities, including tasks that rely on motor learning (Bernard and Seidler, 2012; Reuter-Lorenz and Park, 2010). These findings support the perspective offered by the STAC, which suggests that older adults rely on compensatory neural processes, both at the physiological and functional levels, to sustain cognitive and motor abilities despite age-related decline (Goh and Park, 2009; Reuter-Lorenz and Park, 2014).

These findings are further aligned with broader models of cognitive aging, such as the Hemispheric Asymmetry Reduction in Older Adults (HAROLD, Cabeza, 2002), which emphasize compensatory recruitment and neuroplastic adaptation. Incorporating EEG-based markers into these frameworks may enhance their utility in predicting who benefits most from specific interventions. As we move toward a precision rehabilitation paradigm, integrating neural, cognitive, and behavioral data will be key to designing scalable, evidence-based interventions for aging populations.

Reductions in frontal delta (0.5–4 Hz) activity have been observed following motor training in young adults (Mak et al., 2013; Wong et al., 2013), and aging is associated with decreased delta activity in occipital areas (Babiloni et al., 2006). Delta activity is associated with internal cognitive processing and decision-making. During externally focused tasks, delta power tends to decrease (Giannitrapani, 1971; Babiloni et al., 2017). Other frequency bands also play key roles. Theta oscillations (4–7 Hz), especially in the frontal cortex, are linked to memory, cognitive control, error monitoring, and conflict resolution (Cavanagh et al., 2009; Cohen et al., 2008). In motor learning, theta power often increases in later stages, especially in parietal and frontal regions (Perfetti et al., 2011; Pitto et al., 2011). Alpha rhythms (8–13 Hz), dominant during relaxed wakefulness, are involved in sensorimotor integration and cognitive effort (Crone et al., 1998). Alpha desynchronization is typically associated with improved performance. While some studies suggest alpha power declines with age (Markand, 1990; Klimesch, 1999), others find this decline only in individuals with cognitive impairments (Jelic and Kowalski, 2009; Babiloni et al., 2006). During motor learning, alpha power has been shown to increase in successful learners (Haufler et al., 2000; Karabanov et al., 2012). Increased alpha desynchronization and theta power in older adults may represent greater cognitive effort during learning, consistent with the idea of neural inefficiency or dedifferentiation in aging. These dynamics underscore the shift from automatic to more effortful motor control strategies in older age.

Reduced alpha power over the sensorimotor cortex has been linked to increased cognitive load, and heightened attentional demands, particularly during intensive (massed) practice in visuomotor learning tasks (Studer et al., 2010). This suggests that when the brain is more actively engaged in processing motor tasks, alpha activity diminishes in regions responsible for sensorimotor integration. Additionally, research has shown that lower levels of neural activity across several brain areas can be associated with more rapid motor learning, implying that efficient learning may involve more focused or economical neural processing (Gehringer et al., 2019). In contrast, aging appears to affect brain oscillations differently. An observed increase in beta band power among older adults may be connected to elevated levels of gamma-aminobutyric acid (GABA) transmission, which plays a key role in inhibitory control within the motor system (Gehringer et al., 2019; Heinrichs-Graham and Wilson, 2016). These patterns reflect age-related neurophysiological changes that can influence motor learning and performance.

The mu rhythm (10–13 Hz), overlaps with the alpha band but is focused on sensorimotor areas and is modulated during movement observation, execution, and imagery (Marshall and Meltzoff, 2011; Pineda, 2005). Mu suppression is a reliable indicator of motor system engagement and has been observed during motor learning (Alhajri et al., 2018).

Effective connectivity, functional connectivity or dynamic communication between brain regions, is crucial for understanding motor learning. Studies have shown that strong connectivity, particularly in alpha-band phase synchronization predicts the response amplitude of the distant brain regions effectively connected to M1 (McGregor and Gribble, 2017; Tomassini et al., 2010; Zazio et al., 2021). Increased functional connectivity within task-relevant networks is associated with more efficient motor performance (Heitger et al., 2012). Resting-state connectivity in the alpha and beta bands has been linked to offline learning and motor adaptation (Manuel et al., 2018; Özdenizci et al., 2017). Moreover, prior research reported gamma oscillations (>30 Hz) are linked to higher-order cognitive processes such as attention, perception, and motor control (Uhlhaas et al., 2010). Increases in gamma activity have been observed during motor execution and imagery, and also after training (Crone et al., 1998; Perfetti et al., 2011; Amo et al., 2017). These increases are thought to reflect the engagement of local cortical networks involved in fine-tuning motor commands and integrating sensorimotor information. Furthermore, enhanced gamma activity following motor training suggests a link between motor learning and cortical plasticity. As individuals practice and refine motor skills, the heightened gamma response may represent more efficient neural synchronization and improved functional connectivity in task-relevant areas. This supports the idea that gamma oscillations not only accompany motor actions but may also play an active role in the consolidation and optimization of motor performance over time.

Together, these findings highlight the importance of brain oscillations in motor learning, particularly as they relate to age-related decline and rehabilitation. Incorporating these neural markers into therapy and training programs could lead to more personalized and effective interventions. Future research should continue to explore how these oscillatory patterns interact with motor learning processes, emphasizing individualized aged-related approaches tailored to neural profiles and specific cognitive-motor needs.

The accumulated evidence underscores the complexity and variability of neural mechanisms involved in motor learning. Studies like Wu et al. (2014) and Penalver-Andres et al. (2022) further emphasize the value of examining connectivity patterns and oscillatory traits at rest to predict motor performance outcomes. The integration of EEG-based frequency analysis with behavioral and task-based metrics provides a powerful framework for understanding motor learning across the lifespan. Continued exploration of frequency-specific dynamics especially beta and its interaction with other bands may yield effective strategies for enhancing neurorehabilitation and mitigating age-related motor deficits.

From a clinical perspective, these findings underscore the value of EEG-based assessment for tailoring motor rehabilitation protocols in aging populations. For example, baseline beta power or alpha connectivity could serve as biomarkers to identify individuals who may benefit from slower-paced, distributed practice formats. Furthermore, EEG-guided neurofeedback interventions could enhance specific oscillatory patterns (e.g., increasing alpha or suppressing excessive beta), thereby improving motor learning efficiency. This neuroadaptive approach aligns well with principles of personalized medicine and could be particularly beneficial for older adults facing early-stage motor or cognitive decline.

### 4.1 Strengths and limitations

This systematic review is built on a comprehensive literature search conducted across multiple databases, ensuring a broad and inclusive capture of relevant studies. The predefined inclusion and exclusion criteria were carefully established to enhance the reliability and validity of the findings, allowing for a consistent evaluation of the available evidence. Quality assessment of the studies was rigorously conducted using the ROB2 (Risk of Bias in Systematic Reviews) tool, adhering to established guidelines to minimize bias and strengthen the conclusions drawn. Our review includes a detailed examination of randomized controlled trials and experimental studies that meet the inclusion criteria, ensuring a high level of evidence. Furthermore, collaboration with field experts has contributed additional insights and credibility to the review, enriching the analysis and interpretation of the data. These strengths collectively contribute to the robustness of the review, providing valuable guidance for future basic research, clinical practice in neurorehabilitation, and motor behavior studies.

Despite its strengths, this systematic review has several limitations that should be acknowledged. One of the primary concerns is the heterogeneity among the included studies, which may affect the generalizability of our findings. Variations in study design, sample sizes, assessment methods, and intervention protocols can introduce inconsistencies, making it challenging to draw definitive conclusions across diverse research contexts. Additionally, some studies included in the review had methodological flaws, such as small sample sizes, lack of proper control groups, or inadequate reporting of results, which may impact the overall quality of the evidence. Our review was also restricted to English-language publications from 2008 onwards, potentially excluding relevant studies published in other languages or prior to this period. This language and timeframe restriction may limit the comprehensiveness of our review and overlook important findings from earlier or non-English research. Moreover, while we made extensive efforts to minimize bias in study selection through rigorous screening and predefined criteria, some degree of subjectivity could still influence the interpretation and synthesis of the data. These limitations underscore the need for cautious interpretation of the results and highlight areas for improvement in future research.

In addition, many studies included in this review had relatively small sample sizes and were often underpowered to be able to detect age-related interaction effects. Furthermore, even a selected group of studies used a longitudinal design, making it difficult to draw conclusions related to long-term motor learning or retention. EEG data quality and preprocessing also varied widely, contributing to variability in reported findings. The heterogeneity of EEG feature extraction, from peak frequency to event-related desynchronization and coherence, further complicates cross-study comparison. While our selection process conformed to rigid inclusion criteria, publication bias is always a risk with the use of published data, given that studies with negative results will more likely go unreported.

Moreover, it was very common for data to not be reported or to be incomplete; for example, some studies failed to report key participant demographic (e.g., gender) or it is not possible to know how these missing key participant demographics impacted the conclusion drawn (e.g., Zhang and Fong, 2019). There were also concerns with selective reporting of outcomes (e.g., some studies reported specific outcome measures and do not report on other relevant outcomes, some outcomes that there were indications of adverse outcomes but they were not reported, etc.). This bias raises suspicion around study findings and limits robustness and generalizability study findings. Ultimately, future study should report all data so other researchers can replicate or use these findings to inform practice or implementation. There are some notable limitations in the research found during the course of this extensive systematic literature review. A number of the reviewed articles did not employ randomized controlled trial designs except for the ten studies previously identified as randomized trials and semi randomized and instead relied on experimental approaches that lacked methodological transparency with short sample sizes and short training intervention times even with representing valuable insights (e.g., Yang et al., 2017; Baumeister et al., 2013; Penalver-Andres et al., 2022).

#### 4.1.1 Future directions

Relying on brain oscillations associated with various aspects of motor learning and performance contributes to the application of neuroscientific methods such as neurofeedback and its different types in healthy populations (Onagawa et al., 2023), athletes and highly specialized skills (Afrash et al., 2023; Xiang et al., 2018; Mirifar et al., 2017). For example, sensorimotor neurofeedback as a method used for regulating brain activities at Cz (central) area was revealed to be effective in facilitating motor learning in golfers. Moreover, it was found that neurofeedback training improved the amplitude of sensorimotor at Cz, suppressed alpha at Fz (Frontal), and is recommended to be applied in order to facilitate longer-term motor learning in golfers (Afrash et al., 2023). However, because individuals with extensive expertise often exhibit distinct neural structures and greater neuroplastic potential compared to non-experts (Furuya et al., 2014; Mizuguchi et al., 2019; Nakagawa et al., 2019), it remains uncertain whether findings observed in expert populations can be reliably applied to the general healthy population. The specialized oscillation-based training and experience of experts may lead to unique adaptations in brain function and anatomy, which could influence how they respond to experimental tasks or interventions. As a result, caution is warranted when attempting to generalize results from expert samples to broader, non-expert groups.

Future work should explore real-time EEG-based neurofeedback protocols tailored to enhance the oscillatory patterns most conducive to learning (e.g., boosting frontoparietal alpha or suppressing beta during specific learning phases). The development and validation of mobile, dry-sensor EEG systems open avenues for monitoring brain activity in everyday environments. This would allow for ecologically valid training and assessment, especially important for older adults in home or community settings. Additionally, machine learning models leveraging EEG coherence and power spectra (e.g., via partial least squares regression) could stratify individuals based on predicted learning potential, guiding the selection of interventions that are most likely to succeed. Longitudinal studies incorporating EEG, behavioral, and structural imaging data are needed to track how neural plasticity evolves with age and intervention. This would help disentangle compensatory vs. restorative mechanisms and inform maintenance strategies for cognitive-motor health. Given the demonstrated benefits of dual-task training in older adults, rehabilitation programs should incorporate cognitive challenges alongside motor tasks to enhance generalization and functional transfer, especially for populations at risk of falls or cognitive decline. We further suggest future studies to consider neurofeedback training for motor learning in healthy adults while taking the duration, protocol, number of sessions, demographics, psychological states, potential psychiatric and neurological symptoms, motor performance, and motor functions (e.g., speed, accuracy, power, and dexterity).

As also reviewed by Peng et al. (2024), a promising avenue for upcoming research involves exploring corticomuscular coherence as it relates to sensorimotor learning. In our systematic review, we learned that several studies have pointed to the importance of beta-band oscillations in executing movement, acquiring motor skills, and in the interaction between brain and muscle activity. Notably, beta corticomuscular coherence appears to play a key role in stabilizing muscle force during static (isometric) contractions. However, its relationship with variables such as movement speed and precision remains less clearly defined and deserves closer investigation. Progress in this area may come from analyzing how different brain regions coordinate with muscle groups during diverse motor tasks (Peng et al., 2024). By combining analyses of both corticomuscular and intermuscular coherence, future researchers can better understand the broader neural systems that support motor adaptation and control, such as tool use training, which was measured in our prior research (Jahanian Najafabadi et al., 2023a,b, 2025). Insights from this line of research may contribute to the development of more precise and effective therapeutic interventions for motor-related disorders, such as stroke. In addition, we propose the use of augmented and extended reality technologies as valuable tools to examine how brain activity varies in response to different task demands and environmental conditions that differ from real-world settings (Jahanian et al., 2023c). For example, virtual objects may lack physical weight, or their perceived weight can be manipulated using physical proxies, offering novel ways to study motor control and sensorimotor adaptation under controlled yet ecologically valid conditions.

## 5 Summary and conclusion

In summary, aging influences the neurophysiological patterns of motor learning but does not eliminate the brain's capacity for functional reorganization and skill acquisition. EEG biomarkers particularly in the alpha, theta, and beta bands along with measures of brain connectivity, offer valuable insight into the neural adaptations that occur with practice and aging. Optimizing training approaches (e.g., exergaming, distributed practice, and task difficulty calibration) can unlock latent plasticity in older adults, enabling meaningful gains in both cognitive and motor domains. These findings support a precision-based approach to rehabilitation and lifelong learning, leveraging individualized neural profiles to enhance outcomes across the lifespan.

Our findings indicate that task design is critical: interventions that are multimodal (e.g., exergaming), adaptive, and cognitively engaging produce more robust neural and behavioral changes than static or less interactive approaches. Additionally, practice structure (massed vs. distributed) and task difficulty play pivotal roles in shaping neural efficiency and long-term retention. For older adults, moderate challenge levels and spaced practice sessions appear to offer the best balance between cognitive demand and plastic potential. Moreover, task familiarity and skill consolidation processes differ with age. Older adults show slower neural adaptation and less efficient modulation of oscillatory activity, yet benefit from overnight consolidation and show sustained gains when conditions are optimal. These insights suggest that while the *rate* and *pattern* of learning may differ across the lifespan, the *capacity* for learning remains viable.

From a translational perspective, these findings advocate for precision-based motor training that is informed by individual neurophysiological profiles. By leveraging resting-state EEG and tracking spectral power, coherence, and real-time connectivity patterns over time, clinicians and researchers can better predict which individuals are likely to benefit from specific types of motor training and adjust protocols accordingly. In conclusion, aging does not represent a fixed barrier to motor learning. Rather, it invites a more refined, evidence-based approach that aligns task demands with the learner's cognitive and neural profile. With the integration of EEG biomarkers and personalized training frameworks, we can move toward a new era of adaptive individualized neurorehabilitation, one that promotes resilience, autonomy, and cognitive vitality well into later life.

## Data Availability

The original contributions presented in the study are included in the article/supplementary material, further inquiries can be directed to the corresponding author.
